# Supplementary material for: Modeling enculturated bias in entrainment to rhythmic patterns
Source: PLoS Comput Biol. 2022 Sep 29;18(9):e1010579. doi: 10.1371/journal.pcbi.1010579 (PMC9553061; doi:10.1371/journal.pcbi.1010579)
Supplement: S1 Text — Parameters used in all simulations presented, alongside other relevant configuration details. (PDF) [file pcbi.1010579.s001.pdf]

S1 Text: Simulation parameters.

All code required to reproduce the simulations (and figures) in this manuscript are available at `https://osf.io/tpwfn/`, further to the parameter listing below. Please refer to Methods and S2 Text for descriptions of the parameters listed here.

Experiment 1

Shared parameters: $dt = .001$, $\mu_0 = 0$, $V_0 = .0002$, $\lambda_0 = .01$, $\eta_e = .005$, $\eta_\mu = .01$

European filter configuration (pPIPPET):

$p^{1:1} = .5$, $\{\lambda_i^{1:1}\} = \{.025, \cdots\}$, $\{v_i^{1:1}\} = \{.0001, \cdots\}$

$p^{2:1} = .5$, $\{\lambda_i^{2:1}\} = \{.05, \cdots\}$, $\{v_i^{2:1}\} = \{.0005, \cdots\}$

Malian filter configuration (pPIPPET):

$p^{1:1} = .33$, $\{\lambda_i^{1:1}\} = \{.025, \cdots\}$, $\{v_i^{1:1}\} = \{.0001, \cdots\}$

$p^{2:1} = .33$, $\{\lambda_i^{2:1}\} = \{.05, \cdots\}$, $\{v_i^{2:1}\} = \{.0005, \cdots\}$

$p^{4:3} = .33$, $\{\lambda_i^{4:3}\} = \{.0375, \cdots\}$, $\{v_i^{4:3}\} = \{.00025, \cdots\}$

Experiment 2

Shared parameters: $dt = .005$, $\mu_0 = 0$, $V_0 = .0002$, $\lambda_0 = .01$, $\eta_e = 0$, $\eta_\mu = 0$

Parameters for rhythm categorization (using pPIPPET): $\{\lambda_i^m\} = \{.02, \cdots\}$, $\{v_i^m\} = \{.0005, \cdots\}$

Experiment 3

Shared parameters: $dt = .005$, $\mu_0 = 0$, $V_0 = .0002$, $\lambda_0 = .01$

Parameters for rhythm categorization (using pPIPPET) are the same as in Experiment 2.

Parameters for rhythm tracking (using PIPPET): $\eta_e = .005$, $\eta_\mu = .01$, $\lambda^{max} = .01$, $V^{max} = .01$
