# Supplementary material for: Modeling enculturated bias in entrainment to rhythmic patterns
Source: PLoS Comput Biol. 2022 Sep 29;18(9):e1010579. doi: 10.1371/journal.pcbi.1010579 (PMC9553061; doi:10.1371/journal.pcbi.1010579)
Supplement: S2 Text — Mathematical process for deriving the PIPPET and pPIPPET filters. (PDF) [file pcbi.1010579.s002.pdf]

S2 Text: Derivation of filter equations.

A Bayesian framework for perceptual entrainment to rhythm

1D PIPPET writeup

Snyder [1] provides a partial differential equation describing the evolution of a probability distribution on a continuously stochastically evolving state that drives the emission of point process events. If the evolution of the underlying state is described by a drift-diffusion process with rate 1:

$$d\phi = dt + \sigma dW_t \tag{1}$$

and events are generated at rate $\lambda(\phi)$, then the evolution of the probability distribution $p_t(\phi)$ is described by

$$dp_t(\phi) = \mathcal{L}[p_t(\phi)]dt + p_t(\phi)\left(\frac{\lambda(\phi)}{\hat{\Lambda}} - 1\right) \cdot (dN_t - \Lambda dt) \tag{2}$$

where $\hat{\Lambda} := \mathbb{E}[\lambda(\phi)]$ (with $\mathbb{E}$ denoting expectation under distribution $p_t(\phi)$), dN_t is the increment in the event count over each dt time step (assumed to be either 1 or 0 with probability 1), and $\mathcal{L}$ is the Kolmogorov forward operator associated with (1):

$$\mathcal{L}[p(\phi)] = -\frac{\partial}{\partial\phi}p(\phi) + \frac{\sigma^2}{2}\frac{\partial^2}{\partial\phi^2}p(\phi) \tag{3}$$

Here we project p onto a Gaussian distribution at each time step by matching mean μ and variance V, which is also the projection with minimal KL divergence. We do this by finding the differentials of these moments of p_t and using them to drive the evolution of these two variables:

$$\begin{aligned} d\mu_t =& \mu_{t+} - \mu_t = \int_\phi \phi p_{t+}(\phi)d\phi - \int_\phi \phi p_t(\phi)d\phi \\ =& \int_\phi \phi\left(p_{t+}(\phi) - p_t(\phi)\right)d\phi = \int_\phi \phi dp_t(\phi)d\phi \\ =& \int_\phi \phi\mathcal{L}[p_t(\phi)]dtd\phi + (\hat{\mu} - \mu_t) \cdot (dN_t - \hat{\Lambda}dt) \end{aligned} \tag{4}$$

where we define $\hat{\mu} := \frac{1}{\hat{\Lambda}}\mathbb{E}\left[\phi\lambda(\phi)\right]$, and

$$\begin{aligned} dV_t =& V_{t+} - V_t = \int_\phi (\phi - \mu_{t+})^2 p_{t+}(\phi)d\phi - \int_\phi (\phi - \mu_t)^2 p_t(\phi)d\phi \\ dV_t =& \int_\phi (\phi - \mu_{t+})^2 \left(p_{t+}(\phi) - p_t(\phi)\right)d\phi \\ &+ \int_\phi \left((\phi - \mu_{t+})^2 - (\phi - \mu_t)^2\right)p_t(\phi)d\phi \\ =& \int_\phi (\phi - \mu_{t+})^2 dp_t(\phi) - (\mu_{t+} - \mu_t)^2 \\ =& \int_\phi (\phi - \mu_{t+})^2 \mathcal{L}[p_t(\phi|N_t)]dtd\phi + \left(\hat{V} - V_t\right) \cdot (dN_t - \hat{\Lambda}dt) \end{aligned} \tag{5}$$

where we define $\hat{V} := \frac{1}{\hat{\Lambda}}\mathbb{E}\left[(\phi - \mu_t)^2\lambda(\phi)\right]$.

Integrating by parts (or following [2]), we can calculate the appropriate integrals of $\mathcal{L}[p_t(\phi|N_t)]$, arriving at a general expression for the variational Bayesian filter on phase for point process data:

$$\begin{cases} d\mu_t = & dt + (\hat{\mu} - \mu_t) \cdot (dN_t - \hat{\Lambda} dt) \\ dV_t = & \sigma^2 dt + \left(\hat{V} - V_t\right) \cdot (dN_t - \hat{\Lambda} dt) \end{cases} \tag{6}$$

We complete the derivation by calculating $\hat{\Lambda}$, $\hat{\mu}$, and $\hat{V}$. This proceeds by first deriving a simple expression for the product $p(\phi)\lambda(\phi)$ as a sum of scaled normal distributions.

In the PIPPET generative model, events are generated at rate

$$\lambda(\phi) = \lambda_0 + \sum_{i=1,2,\cdots} \frac{\lambda_i}{\sqrt{2\pi v_i}} e^{-\frac{(\phi-\phi_i)^2}{2v_i}}$$

$p(\phi)$ is assumed (forced) to be Gaussian, so we can write:

$$p(\phi) = \frac{1}{\sqrt{2\pi V}} e^{-\frac{(\phi-\mu)^2}{2V}}.$$

We calculate:

$$\begin{aligned} p(\phi)\lambda(\phi) =& \frac{1}{\sqrt{2\pi V}} e^{-\frac{(\phi-\mu)^2}{2V}} \left(\lambda_0 + \sum_{i=1,2,\cdots} \frac{\lambda_i}{\sqrt{2\pi v_i}} e^{-\frac{(\phi-\phi_i)^2}{2v_i}}\right) \\ =& \frac{\lambda_0}{\sqrt{2\pi V}} e^{-\frac{(\phi-\mu)^2}{2V}} + \sum_{i=1,2,\cdots} \frac{\lambda_i}{2\pi\sqrt{v_i V}} e^{-\frac{1}{2}\left(\frac{(\phi-\phi_i)^2}{v_i} + \frac{(\phi-\mu)^2}{V}\right)} \end{aligned} \tag{7}$$

We next apply a well-known result about quadratic forms that allows us to write a product of Gaussian functions as a Gaussian function:

$$A(x-a)^2 + B(x-b)^2 = \frac{AB}{(A+B)}(a-b)^2 + (A+B)\left(x - \frac{Aa+Bb}{A+B}\right)^2 \tag{8}$$

Applying this result to (7) with $x = \phi$, $a = \phi_i$, $b = \mu$, $A = \frac{1}{v_i}$, and $B = \frac{1}{V}$,

$$\begin{aligned} p(\phi)\lambda(\phi) =& \frac{\lambda_0}{\sqrt{2\pi V}} e^{-\frac{(\phi-\mu)^2}{2V}} + \sum_{i=1,2,\cdots} \frac{\lambda_i}{2\pi\sqrt{v_i V}} e^{-\frac{1}{2}\left(\frac{(\phi_i-\mu)^2}{v_i+V} + \left(\frac{1}{v_i}+\frac{1}{V}\right)\left(\phi - \frac{\frac{\phi_i}{v_i}+\frac{\mu}{V}}{\frac{1}{v_i}+\frac{1}{V}}\right)^2\right)} \\ =& \frac{\lambda_0}{\sqrt{2\pi V}} e^{-\frac{(\phi-\mu)^2}{2V}} + \sum_{i=1,2,\cdots} \lambda_i \left(\frac{1}{\sqrt{2\pi(v_i+V)}} e^{-\frac{(\phi_i-\mu)^2}{2(v_i+V)}}\right)\left(\frac{1}{\sqrt{2\pi\hat{V}_i}} e^{-\frac{(\phi-\hat{\mu}_i)^2}{2\hat{V}_i}}\right) \end{aligned} \tag{9}$$

where we define $\hat{V}_i = \frac{1}{v_i^{-1}+V^{-1}}$ and $\hat{\mu}_i := \hat{V}_i\left(\frac{\phi_i}{v_i} + \frac{\mu}{V}\right)$ for $i > 0$.

These two final terms are both expressions for normal distributions, so we can rewrite (9) as

$$p(\phi)\lambda(\phi) = \lambda_0\varphi(\phi|\mu, V) + \sum_{i=1,2,\cdots} \lambda_i\varphi(\phi_i|\mu, v_i+V)\varphi(\phi|\hat{\mu}_i, \hat{V}_i) \tag{10}$$

We simplify this expression by:

- defining $\Lambda_i := \lambda_i\varphi(\phi_i|\bar{\phi}, v_i+V)$ for $i > 0$, and setting $\Lambda_0 := \lambda_0$
- setting $\hat{V}_0 := V$
- setting $\hat{\mu}_0 := \mu$.

This lets us write

$$p(\phi)\lambda(\phi) = \sum_{i=0,1,\cdots} \Lambda_i \varphi(\phi|\hat{\mu}_i, \hat{V}_i) \tag{11}$$

We use this expression and the moments of normal distributions to calculate $\hat{\Lambda}$, $\hat{\mu}$, and $\hat{V}$:

$$\hat{\Lambda} := \mathbb{E}_p\left[\lambda(\phi)\right] = \sum_{i=0,1,\cdots} \Lambda_i \int \varphi(\phi|\hat{\mu}_i, \hat{V}_i) d\phi = \sum_{i=0,1,\cdots} \Lambda_i \tag{12}$$

$$\begin{aligned}\hat{\mu} :=& \frac{1}{\hat{\Lambda}} \mathbb{E}\left[\phi\lambda(\phi)\right] = \frac{1}{\hat{\Lambda}} \sum_{i=0,1,\cdots} \Lambda_i \int \phi\varphi(\phi|\hat{\mu}_i, \hat{V}_i) d\phi \\ =& \frac{1}{\hat{\Lambda}} \sum_{i=0,1,\cdots} \Lambda_i \hat{\mu}_i \end{aligned} \tag{13}$$

$$\begin{aligned}\hat{V} :=& \frac{1}{\hat{\Lambda}} \mathbb{E}_p\left[(\phi - \mu_{t+})^2 \lambda(\phi)\right] \\ =& \frac{1}{\hat{\Lambda}} \sum_{i=0,1,\cdots} \Lambda_i \int (\phi - \mu_{t+})^2 \varphi(\phi|\hat{\mu}_i, \hat{V}_i) d\phi \\ =& \frac{1}{\hat{\Lambda}} \sum_{i=0,1,\cdots} \Lambda_i \int (\phi - \hat{\mu}_i)^2 \varphi(\phi|\hat{\mu}_i, \hat{V}_i) d\phi + \Lambda_i \int \left((\phi - \mu_{t+})^2 - (\phi - \hat{\mu}_i)^2\right) \varphi(\phi|\hat{\mu}_i, \hat{V}_i) d\phi \end{aligned} \tag{14}$$

The first term is simply the variance of the normal distribution:

$$\begin{aligned} =& \frac{1}{\hat{\Lambda}} \sum_{i=0,1,\cdots} \Lambda_i \hat{V}_i + \Lambda_i \int \left(-2\phi\mu_{t+} + \mu_{t+}^2 + 2\phi\hat{\mu}_i - \hat{\mu}_i^2\right) \varphi(\phi|\hat{\mu}_i, \hat{V}_i) d\phi \\ =& \frac{1}{\hat{\Lambda}} \sum_{i=0,1,\cdots} \Lambda_i \left(\hat{V}_i + (\mu_{t+} - \hat{\mu}_i)^2\right) \end{aligned} \tag{15}$$

Expressions (12), (13), and (15) coupled with (6) constitute the PIPPET filter.

Template selection

When multiple templates are possible, we let $T \in \{1, \ldots, n_T\}$ denote a discrete random variable representing which of n_T possible templates are being used to generate the rhythm. The posterior distribution on T at time t is described by a probability function over the discrete space $\{1, \ldots, n_T\}$ which can be fully characterized by the set of time-varying probabilities $p^m := Pr(T = m)$ for $m = 1, \ldots, n_T$ (which must sum to 1).

Simultaneous phase inference and template probability tracking are handled by performing three consecutive operations each dt time step:

1. The distribution on T is adjusted to incorporate a new observation (the presence or absence of an event at time t) by applying Bayes Rule.
2. The current distribution on ϕ is used to calculate n_T posterior distributions, each conditioned on one of the possible templates, using PIPPET.
3. The n_T posterior distributions are marginalized over templates, and the resulting distribution is approximated by a new Gaussian distribution on ϕ to be used on the next time step.

The rationale of the final step is that entrainment seems to be "single stream" – even professional percussionists report that they cannot track or produce multiple cyclically structured sound streams independently of each other [3, p48]. Thus, multiple possible interpretations of the rhythm can be dynamically weighed against each other, but are not used to maintain independent dynamic estimates of phase – instead, a single estimate is influenced by each template proportionately to its current plausibility.

Step 1: Updating the distribution on T

$$p^m_{t+dt} = Pr(T=m|dN_t)$$

Applying Bayes Rule:

$$= Pr(T=m)\frac{Pr(dN_t|T=m)}{Pr(dN_t)} \tag{16}$$

$$= p^m_t \frac{Pr(dN_t|T=m)}{Pr(dN_t)} \tag{17}$$

$$dp^m := p^m_{t+dt} - p^m_t \tag{18}$$

$$= p^m_t\left(\frac{Pr(dN_t|T=m)}{Pr(dN_t)} - 1\right) \tag{19}$$

The probability $Pr(dN_t|T=m)$ depends on the current phase ϕ, which is known to the observer only as a Gaussian posterior distribution. Thus, the value of this probability is calculated by marginalizing over that distribution:

$$Pr(dN_t|T=m) = \int_\phi Pr(dN_t|T=m, \phi_t=\phi)Pr(\phi_t=\phi)d\phi \tag{20}$$

$$= \int_\phi Pr(dN_t|T=m, \phi_t=\phi)\varphi(\phi|\mu, V)d\phi \tag{21}$$

If there was an event ($dN_t = 1$) then we have

$$Pr(dN_t=1|T=m) = \int_\phi (\lambda^m(\phi)dt)\varphi(\phi|\mu, V)d\phi \tag{22}$$

From (12),

$$= \sum_i \Lambda^m_i dt \tag{23}$$

$$Pr(dN_t=1) = \sum_m Pr(T=m)Pr(dN_t=1|T=m) \tag{24}$$

$$= \sum_m p^m_t \sum_i \Lambda^m_i dt \tag{25}$$

Setting $\hat{\Lambda}^m := \sum_i \Lambda^m_i$ and $\hat{\Lambda} := \sum_m p^m_t \sum_i \Lambda^m_i$ and substituting into (19), we can write

$$dp^m = p^m_t\left(\frac{\hat{\Lambda}^m}{\hat{\Lambda}} - 1\right) \tag{26}$$

If there is no event ($dN_t = 0$) then we have

$$Pr(dN_t=0|T=m) = \int_\phi (1-\lambda^m(\phi)dt)\varphi(\phi|\mu, V)d\phi \tag{27}$$

$$= 1 - \hat{\Lambda}^m dt \tag{28}$$

$$Pr(dN_t=1) = \sum_m Pr(T=m)Pr(dN_t=1|T=m) \tag{29}$$

$$= \sum_m p^m_t \sum_i (1-\hat{\Lambda}^m dt) \tag{30}$$

$$= 1 - \hat{\Lambda} \tag{31}$$

$$dp^m = p^m_t\left(\frac{1-\hat{\Lambda}^m dt}{1-\hat{\Lambda}dt} - 1\right) \tag{32}$$

Using a linear approximation for small dt:

$$dp^m = p_t^m \left(\hat{\Lambda} - \hat{\Lambda}^m\right) dt = p_t^m \left(\frac{\hat{\Lambda}^m}{\hat{\Lambda}} - 1\right)(-\Lambda dt) \tag{33}$$

Since (33) is vanishingly small relative to (26), equations (26) and (33) can be written together as

$$dp^m = p_t^m \left(\hat{\Lambda} - \hat{\Lambda}^m\right) dt = p_t^m \left(\frac{\hat{\Lambda}}{\hat{\Lambda}^m} - 1\right)(dN_t - \hat{\Lambda} dt)$$

Step 2: Calculating a posterior on phase for each template

For each template m, we advance the distribution on ϕ forward by a dt time step using the PIPPET algorithm derived above with $\lambda = \lambda^m$. This results in a set of Gaussian posterior conditioned on each template:

$$Pr(\phi|T = m) = \varphi(\phi|\mu^m, V^m)$$

Step 3: Approximating a sum of Gaussian posteriors with a single Gaussian posterior

The "best" (KL-divergence-minimizing, and hence free energy minimizing) Gaussian approximation of a distribution is the Gaussian that matches the distribution's mean and variance [4]. After the two steps above, the distribution over phase and template can be described as:

$$Pr(\phi, T = m) = Pr(\phi|T = m)Pr(T = m) = \varphi(\phi|\mu^m, V^m)p^m$$

Marginalizing over templates, we have

$$Pr(\phi) = \sum_m p^m \varphi(\phi|\mu^m, V^m) \tag{34}$$

which has mean

$$\mu := \int_\phi \phi Pr(\phi) d\phi = \sum_m p^m \mu^m \tag{35}$$

and variance

$$V := \int_\phi \phi^2 Pr(\phi) d\phi - \mu^2 = \int_\phi \phi^2 \sum_m p^m \varphi(\phi|\mu^m, V^m) d\phi - \left(\sum_m p^m \mu^m\right)^2 \tag{36}$$

The first term represents the second moment of a sum of Gaussians, which is equal to the sums of their second moments $V^m + (\mu^m)^2$, so we have

$$V = \sum_m p^m (V^m + (\mu^m)^2) - \sum_m (p^m \mu^m)^2 - 2 \sum_{m \neq n} p^m \mu^m p^n \mu^n \tag{37}$$

$$= \sum_m p^m V^m + \sum_m p^m (1 - p^m)(\mu^m)^2 - \sum_{m \neq n} 2 p^m p^n \mu^m \mu^n \tag{38}$$

References

[1] Snyder D. Filtering and detection for doubly stochastic Poisson processes. IEEE Transactions on Information Theory. 1972;18(1):91–102.

[2] Eden UT, Brown EN. Continuous-time filters for state estimation from point process models of neural data. Statistica Sinica. 2008;18(4):1293.

[3] London J. Hearing in Time. Oxford University Press; 2012.

[4] Opper M, Winther O. A Bayesian approach to on-line learning. Saad D, editor. Cambridge University Press Cambridge; 1998.
