# Supplementary material for: Modeling enculturated bias in entrainment to rhythmic patterns
Source: PLoS Comput Biol. 2022 Sep 29;18(9):e1010579. doi: 10.1371/journal.pcbi.1010579 (PMC9553061; doi:10.1371/journal.pcbi.1010579)

S1 Fig. Tracking the phase of a 4:3 rhythm with different timing expectations. Two pPIPPET models are given patterns of expectations for 1:1 and 2:1 rhythms, but only one with expectations for 4:3 rhythms. The resulting quality of phase tracking – for the first five stimulus repetitions – is shown through adjustments to estimated phase μ_t on auditory events, alongside changes in uncertainty V_t. Implicit inference of the rhythmic pattern over time is shown through changes in template probability p^m. A) European model. Without 4:3 expectations, phase must be adjusted after the first event of each cycle to compensate for the timing shift, causing phase uncertainty to increase until the cycle is complete, when phase is shifted back. B) Malian model. Phase is successfully tracked, with phase uncertainty only growing slightly between events.

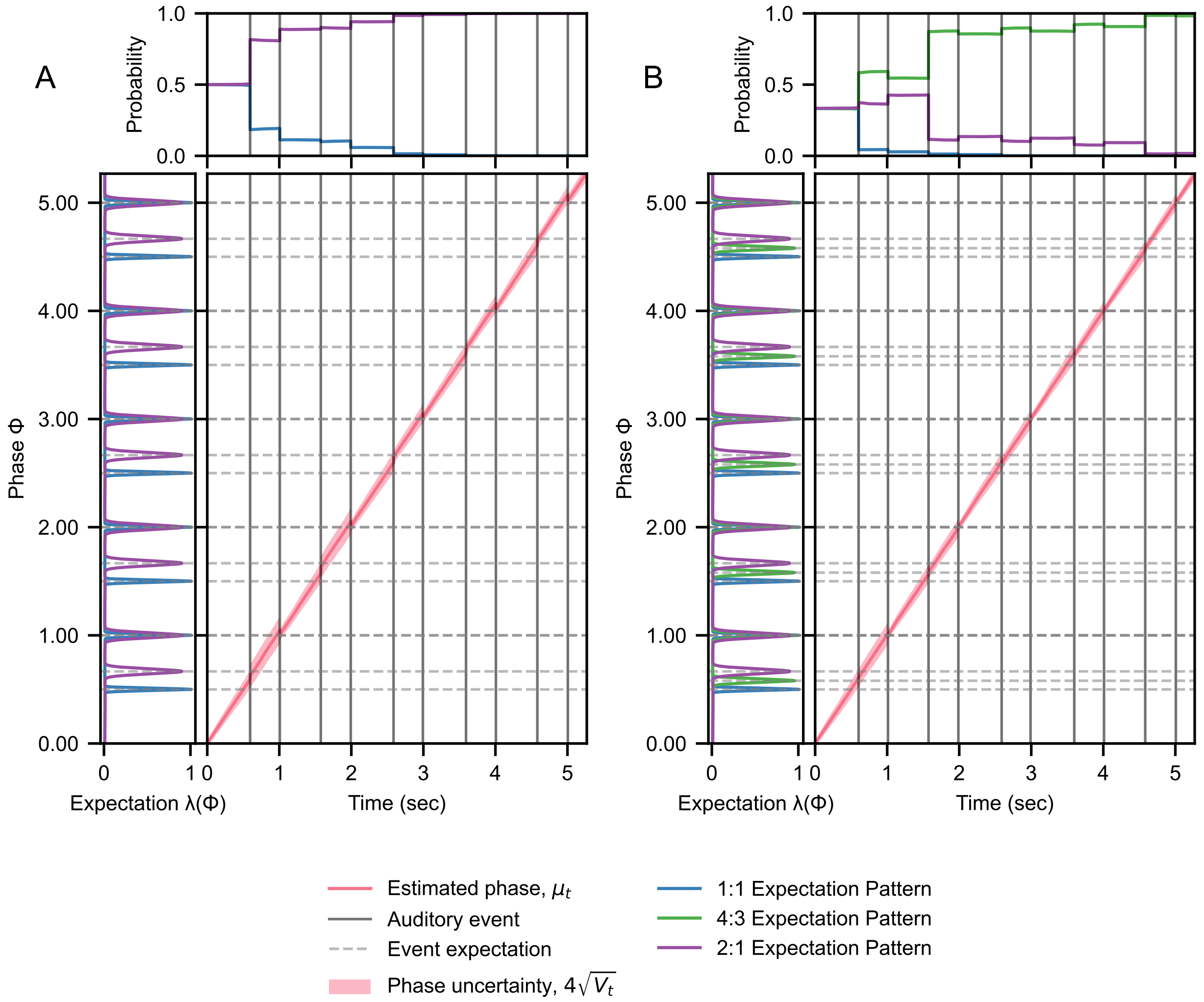

Supplement: S1 Fig — Fig 4 extended to tracking the first five repetitions of the 4:3 rhythm. (PDF) [file pcbi.1010579.s003.pdf]
