# Supplementary material for: Modeling enculturated bias in entrainment to rhythmic patterns
Source: PLoS Comput Biol. 2022 Sep 29;18(9):e1010579. doi: 10.1371/journal.pcbi.1010579 (PMC9553061; doi:10.1371/journal.pcbi.1010579)

S2 Fig. Tracking the phase of a 1:1 rhythm with different timing expectations. Two pPIPPET models are given patterns of expectations for 1:1 and 2:1 rhythms, but only one with expectations for 4:3 rhythms. The resulting quality of phase tracking – for the first five stimulus repetitions – is shown through adjustments to estimated phase μ_t on auditory events, alongside changes in uncertainty V_t. Implicit inference of the rhythmic pattern over time is shown through changes in template probability p^m. A) European model. Phase is successfully tracked, with phase uncertainty only growing slightly between events. B) Malian model. With 4:3 expectations, which are numerically close to the 1:1 stimulus ratio, it takes an additional cycle to infer whether the underyling pattern is a 1:1 or 4:3 rhythm, which temporarily increases phase uncertainty.

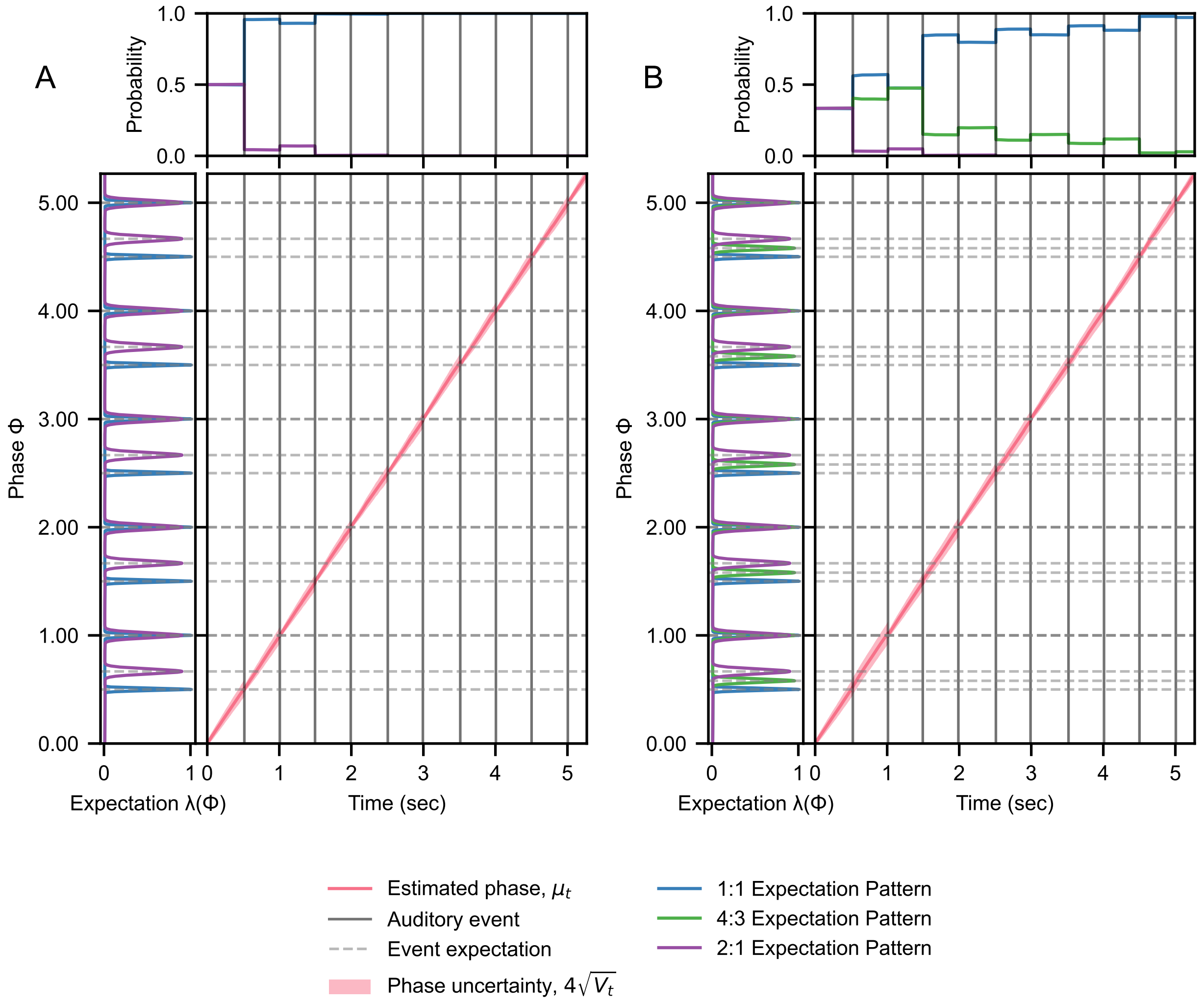

Supplement: S2 Fig — pPIPPET models configured as per Fig 4, tracking the first five repetitions of a 1:1 (isochronous) rhythm. (PDF) [file pcbi.1010579.s004.pdf]
