# Supplementary material for: Modeling enculturated bias in entrainment to rhythmic patterns
Source: PLoS Comput Biol. 2022 Sep 29;18(9):e1010579. doi: 10.1371/journal.pcbi.1010579 (PMC9553061; doi:10.1371/journal.pcbi.1010579)

S3 Fig. Tracking the phase of a 2:1 rhythm with different timing expectations. Two pPIPPET models are given patterns of expectations for 1:1 and 2:1 rhythms, but only one with expectations for 4:3 rhythms. The resulting quality of phase tracking – for the first five stimulus repetitions – is shown through adjustments to estimated phase μ_t on auditory events, alongside changes in uncertainty V_t. Implicit inference of the rhythmic pattern over time is shown through changes in template probability p^m. A) European model. Phase is successfully tracked, with phase uncertainty only growing slightly between events. B) Malian model. In both cases the underlying 2:1 pattern is identified quickly, with likelihood converging (almost) maximally within a single stimulus cycle.

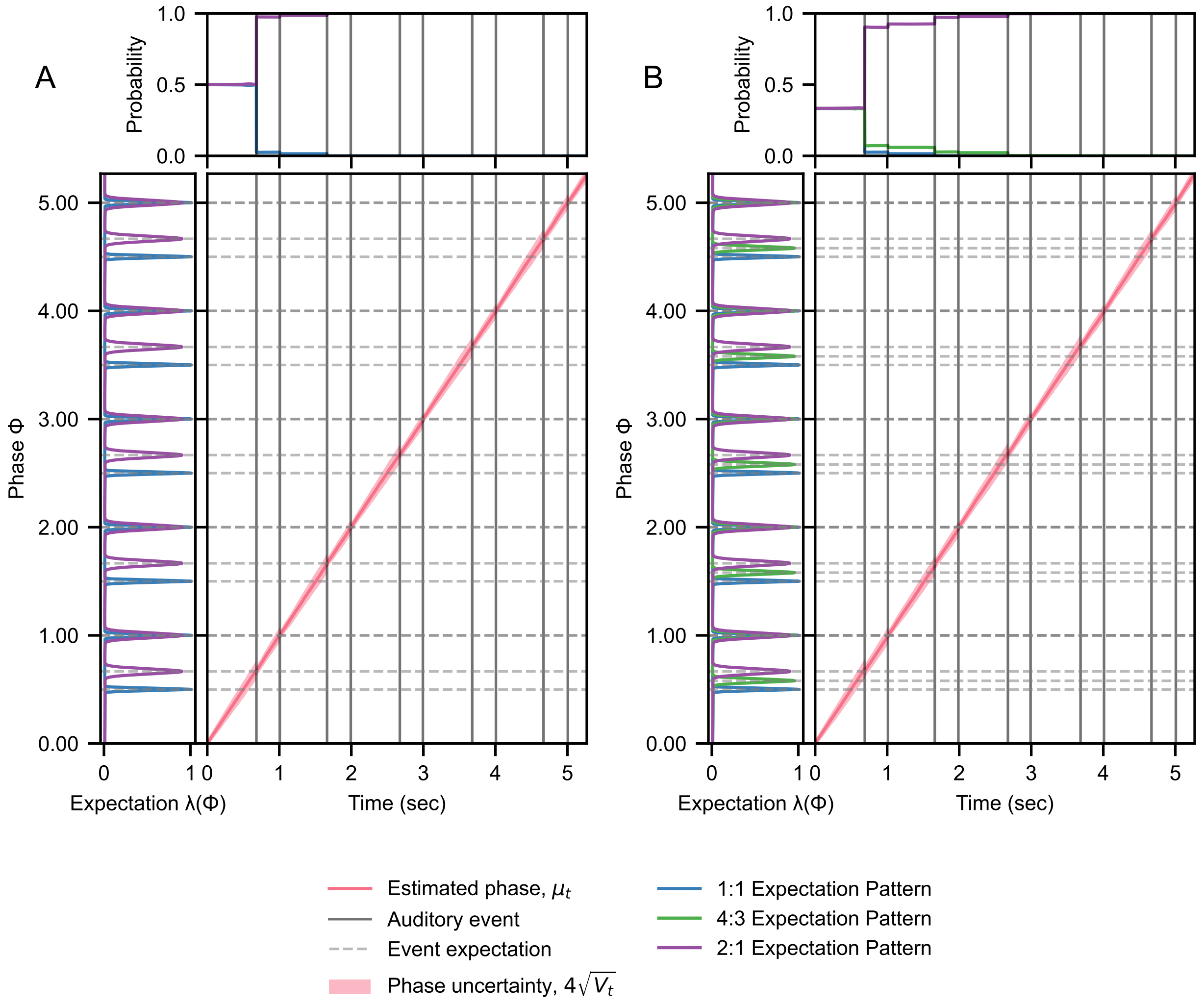

Supplement: S3 Fig — pPIPPET models configured as per Fig 4, tracking the first five repetitions of a 2:1 (uneven) rhythm. (PDF) [file pcbi.1010579.s005.pdf]
