# Supplementary material for: Modeling enculturated bias in entrainment to rhythmic patterns
Source: PLoS Comput Biol. 2022 Sep 29;18(9):e1010579. doi: 10.1371/journal.pcbi.1010579 (PMC9553061; doi:10.1371/journal.pcbi.1010579)

S4 Fig. Expected patterns derived from the German and Turkish rhythm samples. Prior likelihood of three-interval rhythms in Experiments 2 & 3, computed according to the relative onset frequencies of rhythmic events in metrical analysis of German and Turkish corpora (see Methods). Rhythms related by small-integer-ratios (integers less than 3) are denoted by green markers.

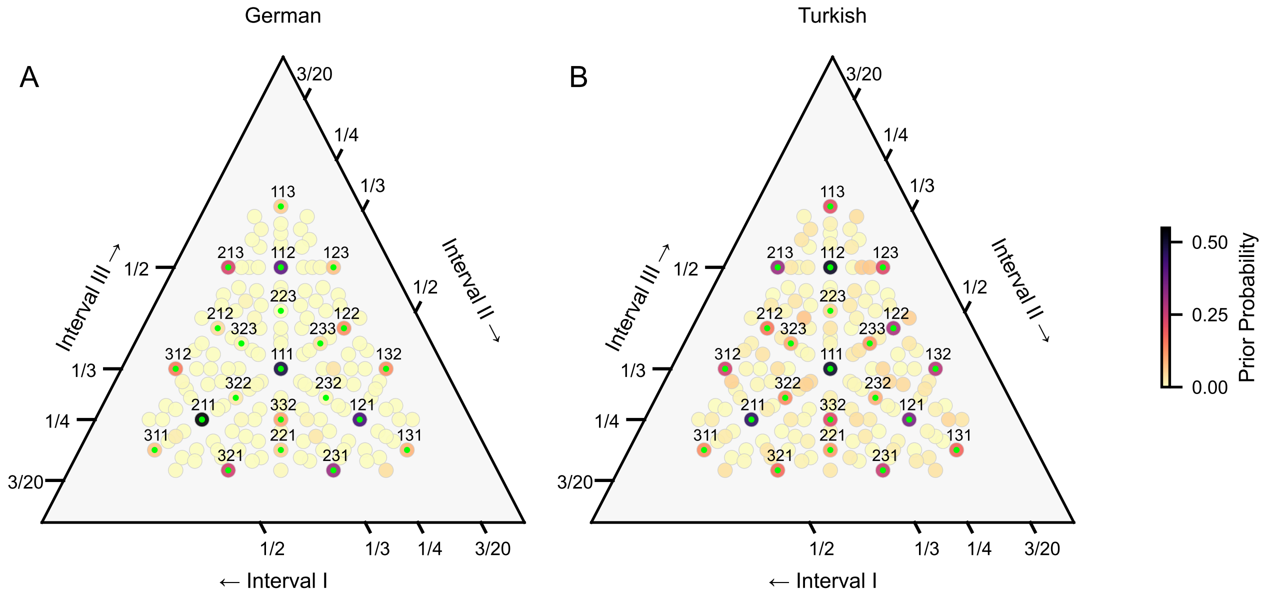

Supplement: S4 Fig — Prior likelihood of three-interval rhythms used to configure the pPIPPET filters in Experiment 2 and Experiment 3. (PDF) [file pcbi.1010579.s006.pdf]
