# Supplementary material for: Modeling enculturated bias in entrainment to rhythmic patterns
Source: PLoS Comput Biol. 2022 Sep 29;18(9):e1010579. doi: 10.1371/journal.pcbi.1010579 (PMC9553061; doi:10.1371/journal.pcbi.1010579)
Supplement: Attachment — Submitted filename: KaplanConnonJamonePearce.pdf [file pcbi.1010579.s007.pdf]

This paper addresses the question of how "enculturated expectations … make certain rhythms easier to track and more conducive to synchronized movement." The paper primarily address the findings of Jacoby and colleagues that 1) Integer ratio "priors" are "universal features of rhythm." And, 2) rhythmic priors are "not fully fixed by biological constraints." The manuscript is generally well written, but I have several concerns, with 1) the loose use to technical terminology, 2) the scholarship of the review section, and 3) the claims about the model and it's implications. I have organized these into three broad sections.

Entrainment:

The authors claim to be studying "enculturated bias in *entrainment*", for which they adopt an empirical definition. They define entrainment as "alignment of perceptual and behavioral processes (e.g. synchronized movement) with regularities in an exogenous stimulus." While a phenomenological definition may be useful to empirical research, as it is agnostic to underlying mechanisms, the question of underlying mechanism is of primary concern to the readers of PLoS Computational Biology, and is main question addressed in this paper. Thus the authors should be extremely cautious with this term.

The term entrainment comes from physics, where it refers to the process of mode locking of coupled driven oscillators. It is a process that leads to temporal coordination, i.e. synchronization, of dynamic events. In neuroscience and psychology, temporal coordination has been observed between sensory signals and neuronal rhythms, and between auditory rhythms and synchronized tapping. It has been termed entrainment because the underlying neural mechanism is generally thought to arise from rhythmic neural oscillations whose phase can be reset by the occurrence of sensory events.

The question of whether the neural process of observed coordination in brain and behavior is oscillatory in the physical sense matters a great deal, and should be of primary concern to the readers of PLoS Computational Biology. Indeed, the authors primary argument seems to be that the mechanism of observed temporal coordination is not oscillatory and therefore, that observed temporal coordination is not really entrainment. This paper argues that the mechanism is, instead, a form of statistical learning. This paper should not be published without a revision that seriously addresses this issue and related terminology.

Review of Previous Literature:

The above issue is especially critical in the domain of musical rhythm perception and production. In the literature on musical rhythm perception, attention, and synchronized action, temporal coordination has been extensively modeled as the coupling of neural oscillations (entrainment). This is a powerful approach because it links rhythmic phenomena such as music, speech, and movement with physical oscillation, a ubiquitous biological mechanism whose predictions are relatively well-understood.

The main prediction of neural oscillation is mode-locking, which predicts a bias toward integer ratios. Such predictions arise through analysis of oscillatory systems in nature, and hundreds of papers have studied mode-locking in many different systems. In previous models of musical rhythm perception, integer ratios are stable dynamical states of synchronizing systems. Predictions of mode-locking are derived by analysis of neural oscillation (Large, Almonte & Velasco, 2011, Physics D). It has been known for nearly 30 years that mode-losing predicts bias toward integer ratios in music perception, production, and synchronization (see, e.g. Large & Kolen, 1994; Lagre & Jones, 1999; Large, 2000).

The current paper takes a quite different, Bayesian approach. The authors posit a filter that dynamically estimates the phase of a periodic process (which, apparently, although it is periodic, is not a neural oscillation). The filter uses an expectation template to inform its estimate of phase based on the presence or absence of events at each time step, pushing it toward expected event phases for each event. The model makes appropriate phase shifts to specific events based prior expectations embodied in the template.

Here is the most important difference from oscillatory models: oscillators (biological or modeled) intrinsically expect integer ratios due to mode locking properties. In the current model, the “prior expectations” are either hand-coded (Experiment 1), or they are learned (Experiment 2) using a statistical approach applied to a corpus of notated rhythms. In the learning model, the likelihood associated with each rhythm is used to configure the prior probability of each expectation a number of expectation templates. Thus, the only reason that the current model “expects” integer ratios is that 1) it has been hand coded to do so, or 2) it has been trained on notated rhythms, which include only integer ratio relationships by definition.

On page 3 the authors state: “Recent psychological experiments have yielded data that are especially challenging for existing computational models of rhythm perception,” citing the two studies by Jacoby et al (discussed below). Which models? At this point in the paper, after the literature review, they have not cited one paper on oscillatory models of rhythm perception, which have been dominant in the field. In fact, they lost completely ignore such models. This is simply not good scholarship.

Regarding the “challenge,” there as been some discussion in the literature (by those not versed in the dynamics of oscillation) that rhythms such as <2,2,3> are not representable by oscillatory models. However, we have shown that an oscillatory model with Hebbian plasticity (another ubiquitous neural mechanism, see, Hoppenstaeadt & Izhikevich, 1996b; Bio Cyb; Kim & Large, 2021, Bio Cyb for the learning models) can learn such “complex” integer-ratio rhythms (e.g., Large Herrera and Velasco, 2015; Tichko & Large, 2019). It was surprising to find that the only reference to this extensive body of work was to one paper in a parenthetical comment in the discussion.

The question is: are integer ratios due to mode-locking, or are they learned based solely on the statistics of the environment? For this paper to be publishable, this question needs to be addressed, not avoided.

Modeling of Jacoby et al’s Findings and related Claims:

Jacoby et al’s findings are two-fold. 1) Integer ratio “priors” are “universal features of rhythm.” And, 2) rhythmic priors are “not fully fixed by biological constraints.” According to J&M, “if priors were themselves tightly constrained, these constraints could in principle explain universal features of rhythm. Previous proposals for such biological and/or cognitive constraints have included oscillatory circuits …”. So Jacoby and McDermott seem to feel that the primary candidate for explaining the universal tendency toward integer ratios is neural oscillation.

As empiricists, they go on to hedge that there have been other proposals. But here’s the thing: Neural oscillation is the only mechanism that has ever been proposed for rhythm perception that is based on a well-known and well-studied neural dynamics. And there is extensive evidence that is consistent with the hypothesis of neural oscillation as the biological basis of rhythm perception and rhythmic attending. And the main prediction of neural oscillation is: integer ratios.

J&M go on to comment that: and “The results leave open the possibility that priors themselves are largely free to reflect whatever distribution occurs in the world.”

The current model takes the this latter approach, but dramatically amplifies it. The current model predicts that priors themselves are completely free to reflect whatever distribution occurs in the world. This manuscript needs to make the point clear.

On the contrary, the manuscript makes the following claim “We demonstrated … perceptual bias towards specific rhythmic patterns during iterated reproduction, including both a universal tendency for simple integer ratio rhythms and culture-specific biases towards rhythmic patterns that distinguish musical cultures. They most certainly did not.

The authors demonstrated a learning algorithm that can capture culture-specific biases for specific rhythmic patterns, that much is true. But this model would learn an*y pattern of temporal intervals, for example* <pi/4, sqrt(2), 1>. Their system only learned integer ratios because they only trained it on integer ratios. They did not demonstrate “a universal tendency for simple integer ratio rhythms.” This paper cannot be published while it includes this claim.

If the authors want to claim that the universal tendency toward integer ratios (as demonstrated by Jacoby et al.) is due to the fact that music around the globe demonstrates a universal tendency for simple integer ratio rhythms, then they should make that argument explicit. But they must make clear that their proposed mechanism demonstrates no such bias.

In such an argument, the authors should deal with the fact that the global study, and a new developmental study found integer ratios only. Nothing else. Surely if any rhythms were learnable there should be one culture in the world, or one developing child that demonstrates a bias for a non-integer rhythm. One comment by J&M is informative: “… the widespread presence of integer ratio rhythms could reflect constraints on production.” To which I would add 1) there is also extensive evidence that constraints on rhythm productions are oscillatory in nature, and 2) virtually all of the the Jacoby experiment are about synchronization / production, 3) Cannon and Patel argue the rhythm perception takes place in motor networks, using rhythm generation circuits for perceptual prediction. These facts, along with the prior theoretical positions of the authors, should be acknowledged and reconciled as well.

Finally, as this manuscript is intended for Computational Biology, the authors should address the biological plausibility of the proposed computational mechanism.
