# Supplementary material for: Modeling enculturated bias in entrainment to rhythmic patterns
Source: PLoS Comput Biol. 2022 Sep 29;18(9):e1010579. doi: 10.1371/journal.pcbi.1010579 (PMC9553061; doi:10.1371/journal.pcbi.1010579)
Supplement: Attachment — Submitted filename: Response_20220506.pdf [file pcbi.1010579.s008.pdf]

Revision of PCOMPBIOL-D-21-02272

Dear Dr Andrea E. Martin, Dr Samuel Gershman and Reviewers,

We are very grateful for the editors' and reviewers' careful reading and detailed comments on our manuscript, which we have systematically addressed in the revised manuscript as detailed in our responses below.

Most notably, we have emphasized that the present work investigates enculturation effects using probabilistic models in which listeners' expectations are learned solely based on the statistics of the rhythmic environment–an empirical sample of musical rhythms from a given musical culture. This raised an important discussion point on the possibility of intrinsic priors (e.g. relating to motor constraints), noted by Reviewers 2 and 3, which we have directly addressed. We do not discount the possibility of such intrinsic constraints, and a new Discussion section considers how constraints might be incorporated in pPIPPET. In this we draw attention to the fact that our computational (and probabilistic) description of entrainment may be compatible with several mechanistic interpretations of entrainment, including oscillatory processes (as highlighted by Reviewer 2). If we were to commit to a specific mechanistic implementation, pPIPPET's priors might be subject to constraints, but this would not fundamentally alter our computational interpretation.

Related to this, in another new Discussion section we make explicit comparisons between pPIPPET and other models (as proposed by Reviewer 3). This includes highlighting the body of work describing rhythmic enculturation effects using oscillatory networks with Hebbian plasticity; which we point out, again, is not mutually exclusive to our approach. A thorough reconciliation of probabilistic and oscillatory accounts of enculturation effects is beyond the scope of the present article but is certainly of interest as we have noted – in fact, some of the authors have an ongoing project exploring this question.

Please find below our responses to the reviewers' comments on our original manuscript. We have labeled reviewer comments (R[1-3]) and author responses (AR-) by point. For example: Reviewer 3, section 1, point 2 (R3.1.2); and the respective response (AR-R3.1.2). As instructed, we have included a version of the manuscript with changes tracked, and all page numbers and line numbers refer to this version.

Comments to the Authors:

Reviewer #1:

R1.0: In their manuscript, "Modeling enculturated bias in entrainment to rhythmic Patterns", the authors present a model of entrainment to time series of events: pPIPPET (an extension of PIPPET, from the same group) which is able to reproduce previously observed universal

and enculturated biases in human entrainment. I consider the paper is well written and the model can help our understanding of how temporal expectations influence entrainment. I only have a few comments.

AR-R1.0: *Thank you, we appreciate the positive feedback. Note that page and line numbers below correspond to the version of the manuscript with tracked changes.*

R1.1: 1.) I think the paper would benefit from reproducing the specific figures from the original studies reporting the results that the current model aims to replicate (if possible and with permission from the authors). This would facilitate the direct comparison and better assessment of the performance of the model.

AR-R1.1: *We completely agree that the paper would benefit from also reporting the results of the human studies that we reference. Unfortunately the data is not publicly available, and the authors of the original studies were not able to send usable data when we requested it.*

R1.2: 2.) Fig 3B, can the authors report statistical results comparing phase uncertainty between the different models and ratios?

AR-R1.2: *We assume you refer here to Fig 5B, which reproduced Fig 3B from Polak et al. (2018). We have reported Welch's t-tests to support our analysis (lines 303-320), and all statistical analyses and results can be found in the supplementary materials (see* `Exp_TwoInterval.ipynb`*).*

R1.3: 3.) Please check that PIPPET and pPIPPET acronyms are used as intended (e.g., line 346 and discussion). Sometimes the constant switch is a bit confusing.

AR-R1.3: *We have ensured the acronyms are used as intended. In certain parts of the Discussion we make a point of referring to PIPPET, as we discuss features/parameters that have not been newly introduced in pPIPPET.*

R1.4: 4.) Throughout the paper, pPIPPET is presented mostly as a model of entrainment. However, the authors state in lines 181-183: "Note that we do not explicitly model behavioral entrainment: instead, we model how the listener would interpret the rhythm in light of their expectations, which we assume would inform their reproduction of the rhythm". Could the authors expand more on this in the discussion? Is the intention to make a distinction between perceptual as opposed to motor entrainment? Could other factors mediating between the interpretation of the rhythms and its reproduction explain for example small differences between the model and empirical data in experiment 3?

AR-R1.4: *Thank you for raising this important question. We have noted in the Overview section that PIPPET is formulated as a model of perceptual entrainment, and by extension pPIPPET is as well (lines 166-171). By applying pPIPPET to explain tapping data, we assume that both perceptual and motor entrainment depend on a common underlying process of phase inference. However, it is possible that additional factors influence motor entrainment - presumably, some differences between the model simulations and human behavior observed in Experiment 3 stem from additional mechanisms producing entrained movement from the phase inference process, alongside effects of sensory feedback from*

movement. We have now addressed this possibility explicitly in the Discussion (lines 585-600).

Reviewer #2:

R2.0: This paper addresses the question of how “enculturated expectations ... make certain rhythms easier to track and more conducive to synchronized movement.” The paper primarily address the findings of Jacoby and colleagues that 1) Integer ratio “priors” are “universal features of rhythm.” And, 2) rhythmic priors are “not fully fixed by biological constraints.” The manuscript is generally well written, but I have several concerns, with 1) the loose use to technical terminology, 2) the scholarship of the review section, and 3) the claims about the model and it’s implications. I have organized these into three broad sections.

AR-R2.0: *Thank you, we very much appreciate your detailed feedback from the perspective of oscillatory systems. We realize this perspective was under-represented in the original manuscript, by oversight, and this has now been addressed in the revised manuscript. Note that page and line numbers below correspond to the version of the manuscript with tracked changes.*

Entrainment:

R2.1: The authors claim to be studying “enculturated bias in *entrainment*”, for which they adopt an empirical definition. They define entrainment as “alignment of perceptual and behavioral processes (e.g. synchronized movement) with regularities in an exogenous stimulus.” While a phenomenological definition may be useful to empirical research, as it is agnostic to underlying mechanisms, the question of underlying mechanism is of primary concern to the readers of PLoS Computational Biology, and is main question addressed in this paper. Thus the authors should be extremely cautious with this term.

The term entrainment comes from physics, where it refers to the process of mode locking of coupled driven oscillators. It is a process that leads to temporal coordination, i.e. synchronization, of dynamic events. In neuroscience and psychology, temporal coordination has been observed between sensory signals and neuronal rhythms, and between auditory rhythms and synchronized tapping. It has been termed entrainment because the underlying neural mechanism is generally thought to arise from rhythmic neural oscillations whose phase can be reset by the occurrence of sensory events.

The question of whether the neural process of observed coordination in brain and behavior is oscillatory in the physical sense matters a great deal, and should be of primary concern to the readers of PLoS Computational Biology. Indeed, the authors primary argument seems to be that the mechanism of observed temporal coordination is not oscillatory and therefore, that observed temporal coordination is not really entrainment. This paper argues that the

mechanism is, instead, a form of statistical learning. This paper should not be published without a revision that seriously addresses this issue and related terminology.

AR-R2.1: *We have used Cannon (2021, PLOS Comp. Bio.)'s empirical definition of entrainment, as here we simulate empirical accounts of entrainment. We have focused on a* ***computational*** *characterisation (i.e. a functional cognitive model) of entrainment in the context of enculturated expectations, as opposed to a specific* ***mechanistic*** *implementation of entrainment (in terms of concrete underlying neural mechanisms). We firmly believe a computational characterisation of enculturation effects on entrainment is of great interest to readers, both in and of itself and in that it can help evidence and motivate mechanistic implementations of entrainment.*

Our primary goal is to answer the question: Can both culturally universal and culturally variable aspects of entrainment be explained by knowledge acquired through experience of a musical culture? To address this question, we develop a probabilistic model of entrainment and rhythmic enculturation, simulating entrainment as dynamic inference about the state of an underlying cyclical process (Cannon, 2021); with respect to enculturated expectations acquired through a process of statistical learning (Pearce, 2018). Our computational implementation may be compatible with a range of mechanistic neural implementations, including oscillatory processes and Hebbian plasticity. An oscillatory implementation might constrain the model's priors or the set of patterns that could be learned, but would not fundamentally alter our computational-level characterisation of enculturation biases in entrainment. These issues are now addressed in a new section in the Discussion (lines 601-622) and our position is made clear in a new paragraph in the Introduction (lines 70-88).

Review of Previous Literature:

R2.2.1: The above issue is especially critical in the domain of musical rhythm perception and production. In the literature on musical rhythm perception, attention, and synchronized action, temporal coordination has been extensively modeled as the coupling of neural oscillations (entrainment). This is a powerful approach because it links rhythmic phenomena such as music, speech, and movement with physical oscillation, a ubiquitous biological mechanism whose predictions are relatively well-understood.

The main prediction of neural oscillation is mode-locking, which predicts a bias toward integer ratios. Such predictions arise through analysis of oscillatory systems in nature, and hundreds of papers have studied mode-locking in many different systems. In previous models of musical rhythm perception, integer ratios are stable dynamical states of synchronizing systems. Predictions of mode-locking are derived by analysis of neural oscillation (Large, Almonte & Velasco, 2011, Physics D). It has been known for nearly 30 years that mode-losing predicts bias toward integer ratios in music perception, production, and synchronization (see, e.g. Large & Kolen, 1994; Lagre & Jones, 1999; Large, 2000).

The current paper takes a quite different, Bayesian approach. The authors posit a filter that dynamically estimates the phase of a periodic process (which, apparently, although it is periodic, is not a neural oscillation). The filter uses an expectation template to inform its estimate of phase based on the presence or absence of events at each time step, pushing it

toward expected event phases for each event. The model makes appropriate phase shifts to specific events based prior expectations embodied in the template.

Here is the most important difference from oscillatory models: oscillators (biological or modeled) intrinsically expect integer ratios due to mode locking properties. In the current model, the “prior expectations” are either hand-coded (Experiment 1), or they are learned (Experiment 2) using a statistical approach applied to a corpus of notated rhythms. In the learning model, the likelihood associated with each rhythm is used to configure the prior probability of each expectation a number of expectation templates. Thus, the only reason that the current model “expects” integer ratios is that 1) it has been hand coded to do so, or 2) it has been trained on notated rhythms, which include only integer ratio relationships by definition.

AR-R2.2.1: *We don’t believe this accurately characterizes the research presented in the paper and have attempted to clarify further in the revised manuscript. First, we have emphasized the research question in the introduction: “The purpose of this research is instead to investigate whether it is possible to simulate listeners’ expectations, including both universal simple integer ratio effects and culturally-acquired deviations, solely through learning the statistics of the rhythmic environment (i.e., without any pre-existing or innate bias for simple-integer ratios).” (lines 82-86). Second, Experiment 1 is clearly introduced as a simple illustration of the basic behavior of the pPIPPET filter (lines 235-237). Third, while it is true that for Experiments 2-3 we used empirical samples of discretized (notated) rhythms to derive priors in our model, it is important to highlight that the samples have an arbitrary distribution of interval patterns, which are certainly not limited to simple and small integer ratio rhythms (see the new S4 Fig, requested in* **R3.8.23***). We address the use of discretized training corpora in the Discussion, where we note that discretization is* ***not*** *necessary for constructing pPIPPET templates (lines 549-559) – this also motivates the Discussion section on possible applications of pPIPPET to modeling expectation patterns that aren’t easily quantizable into integer-ratio intervals (e.g. swing in jazz, lines 709-725).*

R2.2.2: On page 3 the authors state: “Recent psychological experiments have yielded data that are especially challenging for existing computational models of rhythm perception,” citing the two studies by Jacoby et al (discussed below). Which models? At this point in the paper, after the literature review, they have not cited one paper on oscillatory models of rhythm perception, which have been dominant in the field. In fact, they lost completely ignore such models. This is simply not good scholarship.

Regarding the “challenge,” there as been some discussion in the literature (by those not versed in the dynamics of oscillation) that rhythms such as <2,2,3> are not representable by oscillatory models. However, we have shown that an oscillatory model with Hebbian plasticity (another ubiquitous neural mechanism, see, Hoppenstaeadt & Izhikevich, 1996b; Bio Cyb; Kim & Large, 2021, Bio Cyb for the learning models) can learn such “complex” integer-ratio rhythms (e.g., Large Herrera and Velasco, 2015; Tichko & Large, 2019). It was surprising to find that the only reference to this extensive body of work was to one paper in a parenthetical comment in the discussion.

AR-R2.2.2: *Apologies for this oversight, we completely agree that this existing work was under-represented in the previous manuscript. This literature is now highlighted in the Introduction (lines 72-82) and again in the Discussion (lines 646-657). We have adjusted the statement on page 3 to note that this "challenge" applies to probabilistic models, and have clarified the Introduction to reflect the probabilistic perspective taken in this work.*

R2.2.3: The question is: are integer ratios due to mode-locking, or are they learned based solely on the statistics of the environment? For this paper to be publishable, this question needs to be addressed, not avoided.

AR-R2.2.3: *This question is certainly very important and interesting! However, in the present paper, we are not attempting to answer this question. We are investigating whether enculturated biases in entrainment–including those for specific integer ratios–can be explained by a model that solely learns through exposure to a musical culture, as now stated very clearly in the introduction. We see this as an important precursor to addressing the question you pose, hence a complementary perspective (as noted in the Discussion, lines 652-657). Also as we stated in* **AR-R2.1***, and elaborated further in the Discussion, pPIPPET may be compatible with oscillatory processes, if priors in pPIPPET were constrained to reflect mode-locking properties.*

Modeling of Jacoby et al's Findings and related Claims:

R2.3.1: Jacoby et al's findings are two-fold. 1) Integer ratio "priors" are "universal features of rhythm." And, 2) rhythmic priors are "not fully fixed by biological constraints." According to J&M, "if priors were themselves tightly constrained, these constraints could in principle explain universal features of rhythm. Previous proposals for such biological and/or cognitive constraints have included oscillatory circuits ...". So Jacoby and McDermott seem to feel that the primary candidate for explaining the universal tendency toward integer ratios is neural oscillation.

AR-R2.3.1: *Indeed, J&M suggest that neural oscillation is one possible candidate, along with others: recurrent attractor networks (Desain & Honing, 1989), and rule-based rhythm representations built on an isochronous pulse (Povel, 1981). We suggest that another possible candidate is probabilistic learning and prediction, without in any way discarding the other candidates.*

R2.3.2: As empiricists, they go on to hedge that there have been other proposals. But here's the thing: Neural oscillation is the only mechanism that has ever been proposed for rhythm perception that is based on a well-known and well-studied neural dynamics. And there is extensive evidence that is consistent with the hypothesis of neural oscillation as the biological basis of rhythm perception and rhythmic attending. And the main prediction of neural oscillation is: integer ratios.

J&M go on to comment that: and "The results leave open the possibility that priors themselves are largely free to reflect whatever distribution occurs in the world."

The current model takes the this latter approach, but dramatically amplifies it. The current model predicts that priors themselves are completely free to reflect whatever distribution occurs in the world. This manuscript needs to make the point clear.

AR-R2.3.2: *Precisely, we build on this open possibility: that priors themselves are learned according to the distribution that occurs in the rhythmic environment. As noted above, we have clarified in the Introduction (lines 82-86) that this is in fact our position.*

R2.3.3: On the contrary, the manuscript makes the following claim “We demonstrated ... perceptual bias towards specific rhythmic patterns during iterated reproduction, including both a universal tendency for simple integer ratio rhythms and culture-specific biases towards rhythmic patterns that distinguish musical cultures. They most certainly did not.

The authors demonstrated a learning algorithm that can capture culture-specific biases for specific rhythmic patterns, that much is true. But this model would learn an*y pattern of temporal intervals, for example* <pi/4, sqrt(2), 1>. Their system only learned integer ratios because they only trained it on integer ratios. They did not demonstrate “a universal tendency for simple integer ratio rhythms.” This paper cannot be published while it includes this claim.

If the authors want to claim that the universal tendency toward integer ratios (as demonstrated by Jacoby et al.) is due to the fact that music around the globe demonstrates a universal tendency for simple integer ratio rhythms, then they should make that argument explicit. But they must make clear that their proposed mechanism demonstrates no such bias.

AR-R2.3.3: *We agree that this sentence was expressed in an imprecise way, and as suggested have corrected it to relate the simulated biases to interval distributions in the training corpora (lines 486-492). The lack of intrinsic biases for certain rhythmic patterns (e.g. simple integer ratios) in pPIPPET is stated explicitly as part of the new Discussion section (mentioned earlier) on intrinsic rhythmic preferences (lines 602-610).*

R2.3.4: In such an argument, the authors should deal with the fact that the global study, and a new developmental study found integer ratios only. Nothing else. Surely if any rhythms were learnable there should be one culture in the world, or one developing child that demonstrates a bias for a non-integer rhythm. One comment by J&M is informative: “... the widespread presence of integer ratio rhythms could reflect constraints on production.” To which I would add 1) there is also extensive evidence that constraints on rhythm productions are oscillatory in nature, and 2) virtually all of the the Jacoby experiment are about synchronization / production, 3) Cannon and Patel argue the rhythm perception takes place in motor networks, using rhythm generation circuits for perceptual prediction. These facts, along with the prior theoretical positions of the authors, should be acknowledged and reconciled as well.

AR-R2.3.4: *Thank you for raising this comment by J&M. In response to* **R1.4** *we explicitly highlighted that differences in perceptual and motor entrainment might cause our simulations to differ somewhat from their empirical results, given your point (2). Again, the possible constraints relating to a given mechanistic interpretation of entrainment, e.g. oscillatory constraints as proposed in (1), are now addressed in the Discussion. Finally, we certainly*

believe a neural implementation of pPIPPET would engage with motor networks – Cannon & Patel's proposal you mention in (3) has inspired one possible neural implementation of PIPPET (p22 of Cannon 2021), and we develop this in the penultimate section of the Discussion titled "pPIPPET in the brain" (lines 727-790).

R2.4.1: Finally, as this manuscript is intended for Computational Biology, the authors should address the biological plausibility of the proposed computational mechanism.

AR-R2.4.1: *As mentioned above, please see the Discussion section titled "pPIPPET in the brain" (lines 727-790). We would be happy to consider any additions that might improve this section.*

Reviewer #3:

Review of pPIPPET

R3.0.1: The work is the extension of the PIPPET model to allow an additional layer of interference, the incorporation of multiple possible expected rhythmic patterns. The goal is to incorporate culturally-specific priors to model previously observed differences in rhythm categorizations. Overall a well written and very stimulating paper that provides a concrete model implementation that can model numerous recent results on cultural priors in the behavioural reproduction of simple rhythms. This model and these new extensions are exciting!

AR-R3.0.1: *Thank you, we appreciate your careful reading and positive feedback. Note that page and line numbers below correspond to the version of the manuscript with tracked changes.*

R3.0.2: The following are general comments and then more specific comments. There were very few outright problems, and none were severe, so much of this can be read as suggestions to strengthen the impact of the paper. Many of the points raised were (I felt) belatedly addressed in the Discussion, so some of this would have been avoided by a better setup in the introduction.

R3.0.3: The top issues for me were: treatment of the status of preferences for small integer ratios, ad-hoc choices regarding relationship between template priors and precision, and vagueness around perception vs. production.

==Modelling==

=Where does small-ratio preference come from?=

R3.1: PIPPET has no small-integer bias built in and presumably would work equally well with rhythmic templates of arbitrary intervals. Here, the preference for small integer ratios is either assumed (Exp 1) or determined empirically by examining corpora. That is, it is _all_ down to experience but the phase estimation model itself has no constraints. This seems profoundly unphysiological. But maybe not--developmental work has showed that infants may have a 'pre-enculturated' phase in which they can discriminate metrical perturbations in small-integer-ratio patterns not prevalent in their culture. Cultures and sub-genres have rhythmic motifs that deviate essentially from simple integer ratios. However, the primacy of small-integer-ratio rhythmic systems would still seem to suggest there are some biological constraints within the brain that bias perception or production towards simple ratios of intervals. Perhaps it is an open question, but I feel it needs more discussion from the start.

Thus, there seems to be an intrinsic circularity, exemplified by the discussion at the top of p. 15, which acknowledges the brain has 'low-level' intrinsic priors, which are not actually modelled in the present work. It seems instead as if the approach is to take musical notation systems as a proxy for the brain's physiological priors and using music notation to externally impose what should rightly be an intrinsic prior of the model itself (if the model is intended to model cognitive operations). It seems backwards?

There is a nice discussion on p. 15 of extending to music as performed, though this does not solve this dilemma of where the quantal nature of rhythm lives--'out there' or 'in here'? There is further excellent discussion on p. 17 of the possibility that with practice, very subtle patterns can be learned, so this bug is actually a feature…but it still remains that simple ratios are easier to learn and perform, so the difficulty of creating templates deviating from simple ratios is something that ultimately needs to be accounted for.

AR-R3.1: *This "dilemma" is certainly very interesting, though challenging to address within the confines of this manuscript. We have tried to clarify the stance taken in the Introduction (lines 82-88). Essentially, in the simulations (Experiments 2 and 3 especially), we set out to test the hypothesis that entrainment - including both universal simple integer ratio effects and culture-specific deviations - can be simulated purely as a single process of probabilistic learning through exposure to a musical culture. However, we remain open to the possibility of intrinsic priors and in a new Discussion section we draw attention to your important point that (p)PIPPET currently has no intrinsic bias for specific rhythmic templates (e.g. small integer bias), and consider how physiological constraints might be incorporated in future iterations of pPIPPET (lines 602-622). We hope this will facilitate future research on the 'in here' / 'out there' question.*

=Predictions?=

R3.2.1: The danger, then, as it is with any modelling work, is that the model just does what you tell it do to with the choice of priors and parameters. I don't think the work can be so simply dismissed but do feel that the value added of this modelling could be brought out more clearly from the beginning.

One way to do that is to discuss the small-ratio issue more completely. Coming to it only in the discussion makes reading up until then a bit more painful than it needs to be. Another way is to focus on other ways in which this model is fundamentally different from existing models, and thus what it adds. E.g., the very empirical results fit here have already been modelled using Bayesian ideas (e.g., in the early work by Honing, Desain and Sadakata). Where does this modelling effort fit in the space of existing models? There IS a nice discussion of this on p. 16, but it is hardly exhaustive of other modelling approaches. Maybe it's just a matter of preference, but I'd like these issues to be set up from the start. Yet another way to add value is to focus on some existing questions within the field that the model suggests answers to (and others may not). Another is to articulate clear predictions for future empirical studies. There were moves in these directions, but nothing really concrete or elaborated.

AR-R3.2.1: *Thanks for these helpful suggestions. In the Introduction we have emphasized deficiencies in existing probabilistic models, which motivates pPIPPET (lines 24-45). We have also addressed the question of simple integer-ratios directly in the Introduction (lines 70-82). We have created a new Discussion section that relates our modeling efforts (and pPIPPET) to existing work (lines 624-657), which includes the earlier models you mentioned by Sadakata, Desain & Honing. Also, we further developed a hypothesis relating to bistable rhythm percepts (lines 777-790), and an immediate application of pPIPPET in investigating Malian rhythmic prototypes (lines 391-400). Together, we hope this helps bring out the value of pPIPPET.*

R3.2.2: These are suggestions. I think this modelling effort to extend PIPPET is cool, novel (in the sense of explicitly modelling phase and its sophisticated formalization) and certainly worthwhile, but I feel all of these could be strengthened and would enhance the readers understanding and the appeal of the ideas here.

AR-R3.2.2: *Again, we really appreciate the positive feedback, and constructive suggestions!*

==The interrelation of priors and precision==

R3.3.1: A different scheme for picking template prior probability and template precision is used in each experiment…Exp 1 uses different precisions and constant priors; Exp 2 uses fixed precisions and empirically-determined priors; Exp 3 uses inverse correlation between prior and precision. This seems ad-hoc and could stand a more unified presentation, or at least greater justification for the choices made in each simulation (or statements that the results do not essentially depend on the choices made).

AR-R3.3.1: *Thank you for raising this important point. As per your suggestion, we have added statements in the model Overview (lines 227-229) and Discussion (lines 508-511) highlighting that the results do not essentially depend on the configuration schemes used. Instead, the schemes used in this work can be seen as starting points for configuring the various terms in pPIPPET and applying it to explain experimental data.*

Most notably, for Experiment 1 we were unable to derive priors and precisions as in Experiments 2-3, as this requires a large empirical sample of Malian rhythms. While a

corpus of Malian Jembe exists (Clayton et al. 2021, curated by Polak), it is small in scale compared to the others used in Experiments 2-3 (from Van der Weij, 2020), which could result in underfitting. Instead, we set a fixed prior as an illustrative example (to be developed in the later experiments), and had to make some intuitive decisions based on the literature to inform precisions.

The choices made for Experiments 2 and 3 are addressed as part of specific comments you have raised below.

Some specific comments relating to this issue:

R3.3.2: 3.66 uncertainty is the _inverse_ of precision, no?

AR-R3.3.2: *Yes, we have corrected this, thank you.*

R3.3.3: 7.223: This is a critical point, that the 2:1 template was defined with lower precision. The reason for assigning lower precision should be justified more clearly--is it because such a template is less common and not as well 'learned' or something else? It has important implications for the evolution of the model's behaviour, e.g., a low-precision template is better able to attract unevenly timed events and thus drives the predictions of the model more strongly (all other things being equal)--this makes sense but is a little bit paradoxical on its face so it may be worth reminding the reader of the relationship between low precision and higher weighting of new evidence (which incidentally could have been better explained at 4.71).

AR-R3.3.3: *The configured precision of the 2:1 template is indeed important and we agree that this needed more emphasis. The reasoning here was based on the extensive literature highlighting the wide attractor effect for 2:1 rhythms, and we have explained how this follows in predictive terms with respect to the relative precision of 2:1 and 1:1 (as you described, lines 273-280). Also, we tried to clarify the related sentence you raise in the Introduction (lines 99-106).*

R3.3.4: As well as precision, the overall priors on the different templates must influence which one wins out. In the models for Experiment 1 the priors are equal, I believe. How is that motivated? It is natural to expect that a template with greater uncertainty for individual note timings should have a lower overall prior. While there is no such formal requirement mathematically, it has intuitive psychological merit and I think it would be a service to readers to articulate this topic more clearly. (Later, in Experiment 3 at 11.358 you do arrive at exactly this point, indeed linking higher priors and higher precision. So, if that is reasonable, why was it not used for all three experiments?)

AR-R3.3.4: *Experiment 1 was intended to illustrate pPIPPET's basic operation (by example). To that end, for simplicity, we only used a few relevant expectation templates and held the priors constant. Though you are right, of course it is meaningful to vary priors, so we now explicitly justify the approach taken in Experiment 1 and point out that this is the approach taken in the subsequent experiments (lines 260-264).*

R3.3.5: The issue of priors comes up in passing also at 9.252. I'm not really sure why you suggest that model behaviour during the first few repetitions matters so much in determining the Malian 1:1 results of Figure 5B--after all, the 1:1 template quickly wins out.

AR-R3.3.5: *This is a good point, S1 Fig reveals that the 1:1 template wins out over a few repetitions. We have removed this comment from the analysis, as we agree it isn't well motivated.*

R3.3.6: How was the precision of the 4:3 template chosen? It looks simply intermediate between 1:1 and 2:1. Why? Also, for accuracy in exposition, it is worth pointing out that you (like Pollok) used 58:42 as the actual ratio, and explain why, as it is hardly a small integer ratio.

AR-R3.3.6: *We have added a note regarding the 58:42 ratio (lines 253-256), thank you for pointing this out. It is unclear what principled approach could be taken to derive the "4:3" template precision, which is an important consideration for future work. Here, an intermediate value was taken, as you observed. This choice contributes to the relative distribution of phase uncertainty (Fig 5B), which as you point out in* **R3.8.27** *shows a remarkable similarity to that of the empirical results.*

R3.3.7: In experiment 2 priors are found empirically, but precision is held constant across templates. Why, exactly?

AR-R3.3.7: *In Experiment 2, we focus on the effects of variable priors without the interacting effects of variable precision terms – given that both priors and precisions contribute to joint phase and pattern inference. This was intended to simplify interpretation of the simulations by focusing just on the new template likelihood (p^m) terms introduced in pPIPPET. We have added notes on this in the Results (lines 364-367) and Discussion(lines 508-510), and definitely plan to investigate the interaction of these terms in future work.*

==Tempo-dependence==

R3.4.1: The recent work by Polak et al found strong results of enculturation at a fast tempo. The current model is tempo-agnostic, so it would not be able to predict tempo-dependent effects such as found by Polak. This deserves comment. I see it is covered a bit in the discussion.

AR-R3.4.1: *We agree that the role of tempo in Polak et al.'s results deserves an explicit comment, which we have expanded on in the Discussion section (lines 563-568).*

==Perception, Production, Both?==

R3.5.1: It is unclear, throughout, whether the behaviour and model are meant to describe perceptual constraints, production constraints, or (as they may be fundamentally intertwined) both. In various places each is mentioned, but vaguely, so I didn't gain a coherent sense of the authors stance on this issue.

AR-R3.5.1: *We hope this has been addressed by* **AR-R1.4**. *Essentially, we assume in the*

present work that both perceptual and motor entrainment depend on a common underlying process of phase inference. However, we acknowledge the possibility of additional mechanisms being involved in production, considered in more detail in the discussion (lines 585-600).

==Replication==

R3.6.1: It is great service that the simulation code is made available through OSF.

==Contributions==

R3.7.1: The manuscript would be enhanced by describing the contributions of each author.

AR-R3.7.1: *Author contributions have been provided as part of the PLOS Comp Bio submission process, and would be published with the final manuscript, if accepted. We are happy to share these earlier, if requested.*

==Specific points==

=Abstract=
R3.8.1: "inferring a suitable learned pattern" could be clearer. As becomes clearer later, it seems like inferring _which_ previously culturally-learned pattern is most suitable to explain the current input.

AR-R3.8.1: *We have adjusted this slightly, to "...inferring which pattern of event timing expectations is most suitable to predict a stimulus rhythm". We believe this proposal stands in a more general sense in the context of pPIPPET, so omit "previously culturally-learned" here and note it later in the abstract.*

=Introduction=
R3.8.2: Overall, found the introduction a bit woolly--it gets across an idea, but the wording is rather imprecise.

R3.8.3: P2.Line2: The first few sentences could stand reformulating. I think I get the gist of what you are trying to say, that people can abstract prototypical rhythms from variable temporal patterns of musical events (?) but there are too many concepts that are fuzzy: 'temporal stress patterns,' 'vary dramatically,' 'discrete rhythmic patterns'

AR-R3.8.3: *We have tried to clarify these opening sentences.*

R3.8.4: 2.6: unclear what 'which' refers to here--I think you intend it to mean that (what is) complexity depends on experience. Complexity doesn't seem like the right, or needed, term here, because it is culturally relative and can be defined in many ways--or do you have some more absolute pattern-based complexity metric in mind?

AR-R3.8.4: *We have removed the mention of "complexity" and this ambiguous "which" – they definitely aren't needed.*

R3.8.5: 2.10: Throughout, would help to make clear if you are talking about the abilities of untrained or musically trained individuals (or dancers). I.e., implicit enculturation vs. trained.

AR-R3.8.5: *This is a good point to raise – we are simulating rather hypothetical listeners and never address how our model might distinguish implicit and explicit effects of musical experience. In each Results section we briefly note that the participants being simulated are all musicians, but added a note in the Discussion highlighting this and the need to address expertise-related effects in future work (lines 519-525).*

R3.8.6: 2.29: An important point regarding interface between continuous rhythmic space and discrete space that can be (easily) written symbolically. Does this work intend to provide insights on the tendency of attraction to simple interval ratios, for example, which surely is cognitively prior to the ability to notate? This point was expanded upon above.

AR-R3.8.6: *We hope this is addressed in* **AR-R3.1**.

R3.8.7: Surprised, however, to see the discussion at such a high level and not invoking early on the universal tendency towards simple ratios…Certainly the fact of attraction to integer ratios was known well before the Jacoby et al experiments cited in the next paragraph, albeit not studied across so many cultures. A more complete historical view of the literature would be appropriate. Refs 1 and 2 are part of the genesis of this entire approach and would seem more appropriate here than in the rather generic first sentences of the intro. Reading further, it's clear the authors are very familiar with the field, so making that more apparent in the intro would have avoided this criticism.

AR-R3.8.7: *You are right to point out that we have only dipped into the historical literature on the tendency towards simple ratios (related to comments in* **R2.2***). This suggestion is well-taken, but here we focus on presenting pPIPPET, and therefore highlight recent experiments by Jacoby et al. as they exemplify the behavior that cannot be simulated with previous probabilistic models of enculturation. We do not feel it is necessary to provide a thorough and historical perspective of the literature in the confines of this manuscript, but have drawn attention to this literature in the Introduction (lines 70-72).*

=Results=

R3.8.8: It would be interesting to comment on how the models converged after the full 25 repetitions…i.e., did they look substantially the same as after the second repetition? The breadth of the distributions in Fig 5 suggest maybe not…that even in the 4:3 case the phases are being reset differently each repetition…[ah, there is noise added to stimulus times…not mentioned until the methods]

AR-R3.8.8: *We have added a note in the Introduction (PIPPET overview, lines 196-199) about these noise terms, apologies that this important detail was left so late. All model*

likelihoods converge after 5 repetitions and rankings (i.e. relative likelihood order) within 2 repetitions (now mentioned on lines 289-292) – we added S1 Fig to show this for the 4:3 stimulus (previous S1/S2 for 1:1 and 2:1 were renamed to S2/S3).

R3.8.9: 8.235: minor point but speaking about "phase corrections performed for all first intervals" is a little confusing--since phase is corrected by events and there is no explicit concept of interval in the model.

AR-R3.8.9: *We have rephrased this in terms of "the first event in each cycle".*

R3.8.10: 8.242: interesting that you are drawing a link between phase uncertainty in the model and tapping uncertainty in people. I really don't see the connection, since the model has a precise representation of mean phase. I suppose one might postulate that mu and sigma aren't represented explicitly in the brain at all, bur are merely parameters of convenience in the model (I.e., the phase could be represented across an ensemble of ramping/oscillating elements), so a tap would be drawn from some kind of distribution of phases that varies at each time point…?

AR-R3.8.10: *We appreciate that there is not a perfect correspondence between phase uncertainty and tapping noise. However, one way to understand the sigma parameter in the generative model is as an <u>estimate</u> of the accumulating noise in the brain's estimate of stimulus phase, i.e., an estimate of timekeeper noise. When that estimate is large, it is because a lot of time has passed since the last rhythmic event, or else recent events were not well-aligned with precise event expectations. These are identical to conditions under which timekeeper noise will have accumulated, and taps will be noisier.*

Another approach would have been to explicitly simulate timekeeper noise on top of the phase inference process; however, this would require a whole additional set of parameters and assumptions, so it made more sense to us to use uncertainty as a proxy for accumulated noise.

R3.8.11: 9.258: Not sure what this means: "allowing pPIPPET to formally characterize behaviour that have previously been related to perceptual "prototypes" or "categories" for rhythm". There have been prior attempts to model using templates and maybe they should be discussed.

AR-R3.8.11: *We have clarified this to point out our precise probabilistic interpretation of these fuzzy high-level terms ("prototype", "category"). We have addressed related models in* **AR-R3.2.1***, but please do suggest other models deserving discussion.*

R3.8.12: 10.300: Curious about the justification for repeating each stimulus once. Experiment 1 showed that the selection of a template (in a simpler situation) took several stimulus repeats to begin to approach an asymptote in template posterior probability. Surely repeating each stimulus more than once is not very complicated.

AR-R3.8.12: *It is not complicated to repeat the stimulus more than once, but here we used a single cycle for consistency with Experiment 3 (and we justify this in* **AR3.8.15** *with respect to the tractability of pPIPPET). For Experiment 2 we have performed analysis using*

additional cycles, and entropy in template likelihoods generally decreases slightly (as you suspected), especially around the boundaries of centroids. But qualitatively the centroids and respective stimulus categorisations do not differ substantially. We are happy to provide and/or further-develop this analysis if you think it is critical.

R3.8.13: 10.304: Not totally clear what the model provides beyond the varying prior probabilities of different templates. Is the strength of attractor determined entirely by its prior, as suggested?

AR-R3.8.13: *Yes it is, though we appreciate (as you highlight in* **R3.3.3***) that attractor strength would also be affected by relative template precisions. Determining the precise interaction between priors and precisions is an important focus of future work, as noted in* **AR-R3.3.7.**

R3.8.14: 11.321: articulation of the model's ability to "generate specific hypotheses" needs more development.

AR-R3.8.14: *We agree that this was incredibly imprecise, apologies. We have now drawn upon a specific example motivated by Polak et al.'s work (as in Experiment 1, lines 391-400).*

R3.8.15: 11.344: in Experiment 3 using only one stimulus cycle to pick a template seems surprising and needs justification, e.g., was the winning template's probability significantly different from all others--i.e., was it always a clear choice? Did the iterated reproduction data show such rapid convergence to a stable pattern? Does this choice of method over-emphasize initially high-prior templates? If instead, waiting over a longer time span might a lower prior template emerge? Or is that never the case--Is the development of marginal probabilities for each template monotonic? It's hard to generalize from the examples shown in Figure 4, which involved at most 3 templates with equal priors. It would be very useful to see some analysis of the Experiment 3 simulations, not just the final results.

AR-R3.8.15: *As you noted in* **R3.8.12***, Experiment 3 is more complicated than the others, and requires analysis of* ***many*** *trials at a high temporal resolution (due to the nature of the experimental paradigm). The decision to use a single stimulus cycle for pattern inference was for simulation tractability, given the computational demands of configuring pPIPPET with an arbitrary number of expectation templates (see Methods, lines 987-1002). In the Discussion (lines 770-777) we consider the possibility that pPIPPET's pattern inference might reflect a (possibly transient) process of rhythm recognition, deployed before or alongside a (partially) separate process of rhythm continuation.*

It would be very interesting to analyze Jacoby et al's observation that participants (in each iterated reproduction trial) commenced tapping after typically a few clicks (i.e. ~1 cycle) – as you suggested, does this data reveal that participants rapidly converge on a stable pattern? Or, might an inferred pattern change visibly within a trial? One prediction from pPIPPET (which you raise) is indeed that longer periods of pattern inference during a trial would increase representation of low-prior templates in the analysis. We have included this

prediction in the Discussion section (lines 785-790).

R3.8.16: 11.352: the phase 'at each event' in the previous stimulus _cycle_. Does this mean the phase estimate at the timestep prior to the events, or the updated phase at the timestep with the event?

AR-R3.8.16: *This refers to the latter, i.e. updated phase at the timestep of the event, which we have tried to clarify.*

R3.8.17: 13.378: clarify what is meant by "less stratified meter" here

AR-R3.8.17: *We have removed the term "stratified" (referring to a meter being organized distinctly in hierarchical levels), as we appreciate it might be unfamiliar to many readers.*

R3.8.18: 16.528: Very interesting discussion. An issue with sensory updating models is that they tend to negate important timing deviations from the beat (establishing e.g., a laid-back feel) by assimilating them into posterior estimates. This might lead to optimal tracking of 'rhythm' but very much sub-optimal tracking of 'beat/meter' and feel. pPIPPET might offer a solution, to the extent that the agent is perceptually aware of the template.

AR-R3.8.18: *Thank you! Though, we are not sure precisely which models you are referring to by "sensory updating models"? To be clear, we consider two possible accounts of "feel" with pPIPPET. First, time-varying phase asynchronies/uncertainty due to expressive timing deviations from a metronomic template (lines 705-709). Second, inference of a template with specific expressively-timed expectations, e.g. for a laid-back or swing feel (lines 712-725). We have tried to make this clearer, but please do let us know if we can improve this Discussion in any way.*

R3.8.19: 14.422: "using an imprecise pattern of expectations" may be a poor word choice given the specific meaning of precision being part of another parameter group (3). Instead "using an inaccurate/non-matching template pattern" or something like that may be better.

AR-R3.8.19: *We have followed this suggestion.*

R3.8.20: 17.559: ASAP is equally attributed to Patel and Iversen.

AR-R3.8.20: *We have corrected the citation, thank you for this reminder.*

R3.8.21: p. 17: The discussion of potential PFC involvement in templates is very well put together.

AR-R3.8.21: *Thank you!*

=Methods=

R3.8.22: Overall, very clear. It would have aided the reader to know earlier about the various places in which noise was added, e.g., to the event times, as this was not clear in the body

of the paper.

AR-R3.8.22: *An earlier mention of noise terms was addressed in* **AR-R3.8.8.**

R3.8.23: The determination of the meter templates from the probabilities of events at each subdivision of the measure is interesting as it pools across different scales (i.e., 111 could be three sixteenths in a row, three eighths, three quarters). Do you have a sense of how adding constraints might change the overall probability space? It would be very useful to see, in supplementary figures, ternary plots of the derived prior probabilities.

AR-R3.8.23: *This is a very interesting question! We did not explore the effects of possible constraints in detail, but existing work (in explicit metrical inference) has reported biases for short versus long tactus intervals on a fixed metrical grid, relating to the total possible instantiations of any pattern (e.g. in §4.3.3 of Van der Weij, 2020). This is definitely something to keep in mind for future work. Also, to address your suggestion, we have added a new supplementary figure (S4 Fig) with the models' priors on ternary plots.*

=Figures=

R3.8.24: The figures are beautifully clear.

AR-R3.8.24: *Thank you!*

R3.8.25: Figure 4: Why does uncertainty grow throughout the cycle in B, even at t=2 by which point the '4:3' model dominates?

AR-R3.8.25: *This is due to the σ term (Eq 2) in PIPPET, which governs the continuous rate of accumulation of phase uncertainty, and can be considered expected phase noise (for further information, see e.g. Fig 4 of Cannon, 2021). For clarity we have also noted this in the Fig 4 caption.*

R3.8.26: Figure 5: Merely out of curiosity, how might you account for the slightly bimodal nature of the European distribution in A (i.e., the mini shelf on the left of the distribution).

AR-R3.8.26: *This relates to the stimulus cycles in which the likelihood of the 2:1 template has not converged on maximum likelihood, causing asynchrony corrections slightly in the direction of the 1:1 template. A model with only 2:1 expectations does not have this "bimodal" distribution – you can see this when removing '1:1' from* `conf_noprior` *in* `Exp_TwoInterval.ipynb` *and regenerating the Fig 5.*

R3.8.27: Naturally it would be helpful to show the empirical data that this reproduces. However, consulting the paper shows a remarkable similarity that could be discussed more forcefully: not only do you find the large difference for 4:3 but you also capture the greater uncertainty at 2:1 than 1:1 (however, is this simply determined by your templates gaussian standard deviations, which vary in the same manner?). A rather secondary issue: The empirical data shows that the Malian uncertainty is greater for _both_ 1:1 as you reproduce but also 2:1, which you do not. Thoughts? Anyway, gets back to the issue of the interaction

between priors and precision discussed above? It would be good to know how the specific parameter choices led to the fits, and what fell out of the model.

AR-R3.8.27: *We agree that showing the empirical data would be valuable, but unfortunately not all this data is publicly available (as noted in* **AR-R1.1***). However, we are pleased that you recognized the similarity of our results.*

Regarding your second point, we did not interpret this in our reading of Polak et al. (2018)'s results. On p11 they report an ANOVA (over reproductions) and note "Within the 2:1 ratio there was no significant difference among groups"; they do not report a specific t-test for group differences in deviations for the 2:1 ratio; and Fig 3B doesn't point toward big differences between the Malian and all of the "European" group (Bulgarian _and_ German participants). Please do correct us if we are missing anything that needs addressing!

R3.8.28: Figure 6: How might one account for the asymmetries in the classification results: e.g., the 112 catchment area is far more permissive of a range of intervals I and II than III (it is not a blob but is oriented northwest-southeast. Another: there is a distinct class for 233 but not 323. Is this (only) because each stimulus was presented once? What might it tell about the importance of order of intervals and how it interacts with the model? Do these details match experimental results?

AR-R3.8.28: *This is another good question! Currently (p)PIPPET does not infer an arbitrary starting phase within the expectation template (though we are working on this), and consequently the results are sensitive to interval order of each expectation template–which depends on respective interval distributions of e.g. 223 and 323 in the corpus. It is certainly possible that these distributions diverge from empirically measured priors. For example, general symmetry is observed across cyclic permutations, with slight preference for patterns where the final interval is longer (discussed on p33 of Jacoby et al., 2021; also Fig S5 of Jacoby & McDermott, 2017). We address these differences in the Discussion (lines 572-581).*

R3.8.29: Figure 7: It would be useful to explain more clearly why is KDE plotted here, vs entropy in Fig 6? I.e., how is this explained by the differences in task (perceptual categorization vs. iterated reproduction)?

AR-R3.8.29: *As you suspect this is related to task differences – we have now noted that the KDE measures the (new) distribution of rhythms as of the last iteration, biased away from the uniformly sampled stimuli (lines 440-443).*

R3.8.30: Figure 8: Phase might better be labelled note onset position (in 16th notes) here, since elsewhere phase 1 = the end of the pattern.

AR-R3.8.30: *We agree this is clearer, and have adjusted Fig 8 and the caption accordingly.*
