# Supplementary material for: Modeling enculturated bias in entrainment to rhythmic patterns
Source: PLoS Comput Biol. 2022 Sep 29;18(9):e1010579. doi: 10.1371/journal.pcbi.1010579 (PMC9553061; doi:10.1371/journal.pcbi.1010579)
Supplement: Attachment — Submitted filename: KaplanCanonReview2.pdf [file pcbi.1010579.s009.pdf]

The authors have addressed many of my comments, and the comments of the other reviewers, and I think the paper is shaping up nicely. However, the results presented still do not support all the claims made through the paper. This is the most significant shortcoming that needs to be addressed before publication.

Unsupported and/or muddled claims about 'universal' biases:

1) In the abstract, the authors say "we simulate iterated reproduction of three-interval rhythms using models configured with rhythm samples from different cultures, demonstrating both universal and enculturated biases as observed experimentally in listeners from those cultures"

This is not accurate, notated rhythms are not rhythm samples from different cultures, i.e. they are not what listeners learn from. Listeners learn from performed auditory rhythms, which — as is well known — deviate routinely, and significantly from notated values. So the authors are claiming more than is supported by the evidence they provide.

Also, the model does not "*demonstrat[e]* … universal biases …", it *learns* integer ratios because that it is trained on integer ratios. The authors state themselves at many places in the paper that there are no pre-existing or innate biases for integer ratios (what Jacoby & McDermott claim are 'universal' biases).

I would suggest: "… *training with a corpus of notated* (or quantized, really) *rhythms* from different cultures …" " … *learning* both the universal and enculturated biases …". A relatively small change in wording, but one that accurately describes what the paper demonstrates.

2) In lines 70-88 authors state: 'The purpose of the present research is instead to investigate whether it is possible to simulate listeners' expectations, including both universal simple integer ratio effects and culturally-acquired deviations, solely through learning the statistics of the rhythmic environment (i.e., without any pre-existing or innate bias for simple-integer ratios)."

(First, note how this statement is not consistent with the claim made in the abstract.)

The answer to this question is 'Yes, if you train with only integer ratio stimuli.' It still seems that the authors are trying to obscure this aspect of their results. But really, the model makes this strong prediction: The reason people demonstrate integer ratio biases is because they experience only (or mostly) integer ratio stimuli. If the authors believe in this approach, they should state this claim plainly.

Also, the phrase " learning the statistics of the rhythmic environment" is not accurate either, because, as pointed out above, the natural rhythmic environment rarely includes quantized rhythms (unless you grow up in a dance club). And the phrase "without any pre-existing or innate bias for simple-integer ratios" implies that such biases will be discovered in the natural rhythmic environment … but the authors used notated rhythms.

3) It is worth pointing out that, despite some suggestions to the contrary toward the end of the paper (see, e.g., lines 549 - 558), the authors have shown no evidence whatsoever that integer ratio biases would be learned by an approach such as theirs when exposed to a natural rhythmic environment. On line 554-556 the authors state that "there is no reason why pPIPPET templates need to be configured with expected events related by perfectly discretized intervals. An alternative approach to configuring pPIPPET might involve analysis of performed (non-quantized) music …" The authors seriously understate the complexity of this issue. Whether or not this approach could learn integer ratio biases when exposed to a natural rhythmic environment is an open question that would require significant future research. The authors should acknowledge this as on serious question, not imply that it is trivial.

In other places in the paper, the authors seem to have their cake and eat it too. In experiment 1, they nicely show that their algorithm can learn a 4.14:3 ratio. In fact, it could learn any ratio. So why not be clear about this in the abstract, conclusions, and throughout the paper. The clear prediction of the model is that humans can learn any rhythmic pattern equally well. Except, … on lines 610 - 612 "However, we don't discount the possibility of intrinsic (innate) priors which could be incorporated into the model." This is frustrating, as the authors seem unwilling to take a theoretical stance. It seems that they don't want to admit any limitations.

Finally, in the two paragraphs beginning on line 602, the authors clarify that it doesn't matter whether a 'bias' is 'universal' or 'learned'. If it turns out that a specific "mechanistic model" shows a universal bias, then they will just change their model to include integer ratio priors. So now I'm perplexed. Isn't it the role of a theory to make predictions about important issues like this?

I suggest one paragraph at the end that straightforwardly and clearly states the findings of this modeling work, the limitations and shortcomings, and the challenges for future research.

Suggestions for consonance with oscillatory approaches (lines 646-657) are interesting. But I don't think top-down vs bottom-up is the issue. The oscillator approach, while respecting the dynamics at the physiological level, is formulated at the behavioral level (e.g., the canonical model; Large, Almonte, & Velasco, 2010). The approach says: if you consider the dynamics of cortical oscillation and Hebbian learning at the physiological level, here are the predictions at the behavioral level. And integer ratio resonances intrinsic to coupled neural oscillation, there's really no getting around that. So, combining the Bayesian approach with the oscillator approach would be more about combining categorization and entrainment, I would think.

Characterization of the oscillation approach

The revision now acknowledges neural resonance theory and its oscillation-based models. But there are some misunderstandings and mischaracterizations in the current manuscript that need to be cleared up. For example, on lines 72-74, the authors attribute the following claim: "This tendency [to … simple integer ratios] might reflect biological constraints on rhythm processing. For example, these simple ratios offer stability for coupled neural oscillators given intrinsic mode-locking properties."

This is almost correct. But NRT does not deal with rhythm 'processing.' NRT claims that the brain resonates to exogenous rhythms. The mode-locking in our theory happens at the the neural level. It does not predict a biological constraints toward perfect integer ratios at the perception or production (ie rhythm processing) level (see even Large & Kolen, 1994, Fg 3 & 6A).

Biological constraints is a (somewhat derogatory) term that has appeared in the literature, used by those who wish to incorrectly attribute limitations to NRT — such as 'oscillator models can only predict things about isochronous stimuli'. Rather than suggest that oscillator models predict 'biological constraints on rhythm processing' it would be more accurate to say that 'this tendency might reflect the physiological dynamics of oscillatory circuits.'

Additionally, (line 76) the authors claim that "Cross-frequency coupled oscillator models … were unable to show enculturated biases for specific ratios." But both of these papers focused on explaining integer ratio categorization, but neither attempted to explain enculturation … and neither even included a learning rule. I would say, 'neither offered an explanation for enculturation.'

Second, as mentioned above, ours is not an 'implementation-level' model. So (line 80) rather than say 'enculturation effects in entrainment might be explained using such (low-level) neural

mechanisms', how about '.. might be explained using a model based on the dynamics of neural oscillation and Hebbian learning.'

On lines 80 - 81 "it is unclear whether these results would generalize to the cultural differences observed in iterated reproduction studies." This conflicts with the statement on lines 654-657 "One interesting point of convergence is in the experience-dependent learning mechanisms proposed – implicit statistical learning might follow the very associative principles of Hebbian learning [68]." How about 'it has not been shown that these results generalize … .'

Issues with Experiment 1:

On lines 273-275, the authors state that "While the timing of stimulus events is closer to a 1:1 rhythm numerically, the stimulus events are attributed to the uneven 2:1 template as the 2:1 template is configured with a lower precision than the 1:1 template. The specificity (high precision) of the 1:1 expectations causes unevenly timed rhythms (e.g. 4:3) to be associated with the comparatively imprecise 2:1 expectations. We liken this effect to the widely observed tendency of listeners to associate uneven rhythms with a long-short pattern (i.e. 2:1)."

But aren't the authors simply building in the behavior that they want to see, because it would not otherwise emerge from the model given the principles they have specified?

More importantly, in the caption to Figure 5 and in lines 300-322 the authors claim that they have 'reproduced' the data from Polak et al. However, if that claim is to be made, they should include the Polak data in this paper, and measure the fit quantitatively. Otherwise, they should temper the claims to something like 'shows qualitative agreement.'

Discussion:

The authors state that "We demonstrated … perceptual bias towards specific rhythmic patterns during iterated reproduction. This bias reflects the interval distributions of music in the training corpora, which exhibit a universal tendency for simple integer ratio rhythms alongside culture-specific biases towards rhythmic patterns deviations from simple integer ratios."

This statement is getting closer to the type of summary that should be included. However, the statement "the interval distributions of music in the training corpora … exhibit a universal tendency for simple integer ratio rhythms … " is not accurate. The interval distributions did not exhibit a universal tendency, the training corpora included only quantized (IR) ratios. Only performed rhythms could — in principle — exhibit a universal tendency. The authors should revise this statement to reflect this.

Also, the training corpora did not exhibit "culture-specific biases towards rhythmic patterns deviations from simple integer ratios." If the authors mean that the model (not the training corpora can exhibit): "culture-specific biases towards rhythmic patterns deviations from simple integer ratios," then the statement should be rewritten to say this. Also, for transparency, this paragraph should clearly state that, in fact, the model will learn whatever patterns it is trained on.

lines 612-614: "We have shown that present empirical results can be accounted for as a process of probabilistic learning. Presumably, musical systems serve as a proxy for priors if they incorporate innate perceptual biases and production constraints [54]." Again, I don't think this is clear. I think it means: We have shown that present empirical results can be accounted for if the musical training material consists only of integer ratio rhythms."

Finally, in lines 739-742 the authors state that "Notably, the authors highlight that this model differs from oscillator-based models with limit cycle regimes, in that the striatium actively shapes SMA activity cycle-by-cycle [84, p10] – which might be better likened to a resetting

drift-diffusion model [85].” First, cycle-by-cycle interaction is how coupled oscillators work, so 84 is not a well-informed counter-argument to oscillation. And [85] was published in 2017. But in 2019, Merchant (PLoS biology) showed oscillatory trajectories in medial premotor cortices (MPCs; pre-supplementary motor area [preSMA] and supplementary motor area [SMA].

Continuing problem with definition and usage of ‘entrainment’:

Rather than acknowledging the physical definition of entrainment, from which the use of this term derives in this field, they have chosen to provide an empirical definition — a definition which is used in empirical work by those who are empirically investigating the phenomenon and necessarily must refrain from assuming the answer to the question they ask. As I have already commented, I don’t believe this is appropriate for a theoretical paper, in which an alternative mechanism is proposed. Or perhaps, since the model is about synchronizing a periodic process with a stimulus, it may be about the physical entrainment, in which case the authors would be claiming our understanding of the physics of entrainment to be revised.

Miscellaneous comments:

In lines 24 - 37 the authors the authors state the there is a history (by the way, it is a *recent* history … not a long tradition, perhaps this could be made clear) of simulating perception and production of rhythm as dynamically mapping between continuous auditory rhythms and discrete symbolic representations. Is this the problem the authors are trying to solve? If not, please be clear that this is a conceptual difficulty with the theory, not merely a problem with the model.

In lines 60-62 the authors state that “There was also substantial variation in the perceptual categories (rhythmic patterns) measured, which was *clearly* related to the prior music-cultural experience of participants.” This is an overstatement. This paper shows a relationship for two of the 39 participant groups. A more accurate formulation would be that they showed ‘some evidence.’
