# Supplementary material for: Modeling enculturated bias in entrainment to rhythmic patterns
Source: PLoS Comput Biol. 2022 Sep 29;18(9):e1010579. doi: 10.1371/journal.pcbi.1010579 (PMC9553061; doi:10.1371/journal.pcbi.1010579)
Supplement: Attachment — Submitted filename: Response_20220823.pdf [file pcbi.1010579.s010.pdf]

Revision of PCOMPBIOL-D-21-02272R1

Dear Dr Samuel Gershman and Reviewers,

We are grateful for the reviewers' further detailed comments on our manuscript, and hope Reviewer 2's remaining concerns are addressed in the newly revised manuscript. We believe that many of these concerns can be addressed by explicitly clarifying two important conceptual distinctions.

First, it is important to distinguish the difference between integer and non-integer ratio rhythms, on the one hand, and the difference between low and high integer ratio rhythms on the other. In training pPIPPET on transcribed music with integer ratio rhythms, we focus on the latter distinction rather than the former (although as we note, pPIPPET can handle both integer and non-integer ratio rhythms). In more detail:

- *Integer vs non-integer ratio rhythms:* As the reviewer rightly points out, much of the music that listeners hear is likely to be performed, hence expressively timed with rhythms that deviate from integer ratios. It has been proposed that listeners categorize such rhythms into integer ratio rhythms at some point during the pipeline of perceptual processing (Clarke, 1987; Desain & Honing, 2003; Honing, 2013). By training pPIPPET on integer-ratio rhythms, we did not intend to address how this categorization process is developed in the course of learning from performed rhythms – as the reviewer highlights, however, this is a non-trivial and interesting question for independent research.

- *Low vs high integer ratio rhythms:* Previous researchers have proposed a perceptual bias towards low-integer ratio rhythms. For example: "there are larger areas around small integer ratios and smaller ones for more complex patterns, indicating more 'tolerance' or freedom in expressive interpretation for rhythms made up of small integers." (Desain & Honing, 2003, p. 347); "To test whether discrete modes at small-integer ratios are universal across groups and to assess the nature and extent of cross-cultural variation, we ran the experiment in a large number of participant groups around the world ... we found small-integer ratios to emerge from the data in each participant group. Small-integer ratios thus indeed appear to be a prominent feature of the way most humans hear music." (Jacoby et al., 2021, pp. 4, 19). It is this tendency towards (simple) low-integer ratio rhythmic interpretations, and cultural variation in preference for specific integer ratios, that is the target of our simulations.

Second, it is important to note that universality need not imply innateness, so when we say universal bias, we mean a bias that is universal across cultures, no more no less. We think the question of what underlies (especially at the physiological level) the universal bias towards simple vs complex integer ratio rhythms is unresolved. It is not that we are unwilling to take a theoretical stance, we just don't think the existing empirical evidence justifies a theoretical position. Rather, we seek to provide evidence that can address the question. In the present paper, we introduce a new model and ask whether a single learning mechanism can account for both the universal bias for low-integer ratio rhythms and culture-specific

variation. In future work, we propose to compare this model with other models in which the universal bias is simulated in different ways, either through priors or through oscillatory dynamics.

Additionally, the reviewer presents the statement “the model will learn whatever patterns it is trained on” as a criticism of the model. We offer two perspectives on this. First, it is not necessarily the case that the model itself will learn whatever patterns it is trained on - every learning mechanism has inductive biases and representational constraints on what it can learn. The question is whether these biases and constraints correspond to those shown by human listeners. Second, the results of Polak et al. (2018) and Jacoby et al. (2021) indicate that listeners across cultures also show evidence of learning the patterns that they are trained on. We think the fact pPIPPET simulates these empirical phenomena provides evidence in its favor.

Please find below our responses to the reviewers’ comments on our revised manuscript. We have labeled reviewer comments (R[1-3]) and author responses (AR-) by point. For example: Reviewer 3, section 1, point 2 (R3.1.2); and the respective response (AR-R3.1.2). As instructed, we have included a version of the manuscript with changes tracked, and all page numbers and line numbers refer to this version.

Comments to the Authors:

Reviewer #1:

R1.0: The authors addressed all my comments. I am happy to recommend the paper for publication

AR-R1.0: *Thank you for your positive and constructive feedback in the previous round of reviews.*

Reviewer #2:

R2.0: The authors have addressed many of my comments, and the comments of the other reviewers, and I think the paper is shaping up nicely. However, the results presented still do not support all the claims made through the paper. This is the most significant shortcoming that needs to be addressed before publication.

AR-R2.0: *Thank you, we appreciate your positive feedback and further detailed comments. Your suggestions have been valuable for improving the accuracy and clarity of the arguments made in the manuscript. Note that page and line numbers below correspond to the version of the manuscript with tracked changes.*

Unsupported and/or muddled claims about ‘universal’ biases:

R2.1.1: 1) In the abstract, the authors say “we simulate iterated reproduction of three-interval rhythms using models configured with rhythm samples from different cultures, demonstrating both universal and enculturated biases as observed experimentally in listeners from those cultures”

This is not accurate, notated rhythms are not rhythm samples from different cultures, i.e. they are not what listeners learn from. Listeners learn from performed auditory rhythms, which — as is well known — deviate routinely, and significantly from notated values. So the authors are claiming more than is supported by the evidence they provide.

Also, the model does not “*demonstrat[e]* ... universal biases ...”, it *learns* integer ratios because that it is trained on integer ratios. The authors state themselves at many places in the paper that there are no pre-existing or innate biases for integer ratios (what Jacoby & McDermott claim are ‘universal’ biases).

I would suggest: “... *training with a corpus of notated* (or quantized, really) *rhythms* from different cultures ...” “ ... *learning* both the universal and enculturated biases ...”. A relatively small change in wording, but one that accurately describes what the paper demonstrates.

AR-R2.1.1: *We agree that this phrasing is more accurate, and have clarified this part of the abstract accordingly.*

R2.1.2: 2) In lines 70-88 authors state: ‘The purpose of the present research is instead to investigate whether it is possible to simulate listeners’ expectations, including both universal simple integer ratio effects and culturally-acquired deviations, solely through learning the statistics of the rhythmic environment (i.e., without any pre-existing or innate bias for simple-integer ratios).”

(First, note how this statement is not consistent with the claim made in the abstract.)

The answer to this question is ‘Yes, if you train with only integer ratio stimuli.’ It still seems that the authors are trying to obscure this aspect of their results. But really, the model makes this strong prediction: The reason people demonstrate integer ratio biases is because they experience only (or mostly) integer ratio stimuli. If the authors believe in this approach, they should state this claim plainly.

Also, the phrase “ learning the statistics of the rhythmic environment” is not accurate either, because, as pointed out above, the natural rhythmic environment rarely includes quantized rhythms (unless you grow up in a dance club). And the phrase “without any pre-existing or innate bias for simple-integer ratios” implies that such biases will be discovered in the natural rhythmic environment ... but the authors used notated rhythms.

AR-R2.1.2: *First, we hope that the changes made as part of* ***AR-R2.1.1*** *improve the consistency of this statement with the abstract. Second, it is not our intention to obscure this aspect of our results, so we have made it clear that training stimuli are notated, i.e. consist of integer ratio rhythms (lines 82-84, 348-451, 484-485, 591-593); and that we focus on the distinction between simple and complex integer ratio rhythms rather than between integer*

and non-integer ratio rhythms (lines 75-82).

Importantly, the training corpora (consisting of real-world notated musical rhythms) do not solely consist of simple-integer ratios but also contain complex integer ratio rhythms (reported in S4 Fig). The question is whether similarities and differences in rhythm perception/production between cultures can be accounted for by similarities and differences in the occurrence of simple and complex-integer ratios between the music of these cultures, together with a model capable of learning these occurrences. If simple integer ratios are common across cultures, the model (like listeners) should exhibit cross-culturally universal tendencies towards simple integer ratio rhythms while deviations towards particular complex ratio rhythms in particular cultures should produce culture specific biases (both in listeners and the model). We added a new paragraph addressing this issue, through the revisions detailed above (lines 75-86).

R2.1.3: 3) It is worth pointing out that, despite some suggestions to the contrary toward the end of the paper (see, e.g., lines 549 - 558), the authors have shown no evidence whatsoever that integer ratio biases would be learned by an approach such as theirs when exposed to a natural rhythmic environment. On line 554-556 the authors state that “there is no reason why pPIPPET templates need to be configured with expected events related by perfectly discretized intervals. An alternative approach to configuring pPIPPET might involve analysis of performed (non-quantized) music ...” The authors seriously understate the complexity of this issue. Whether or not this approach could learn integer ratio biases when exposed to a natural rhythmic environment is an open question that would require significant future research. The authors should acknowledge this as on serious question, not imply that it is trivial.

AR-R2.1.3: *This is certainly a non-trivial extension of this work, and we now explicitly state this (line 541-548). But, depending on the definition of “natural rhythmic environment”, we think some approaches might not be so complex. For example, as a first step, the probability density of specific interval ratio patterns can be calculated from non-quantized rhythmic stimuli using kernel density estimation (in much a similar way to that of Roeske et al., 2020). Assuming a sufficiently large training corpora was available, this might replace the approach described in our Methods (lines 922-972). Generalizing this approach to acoustic representations of natural polyphonic music, in a dynamic context, would be a challenging but worthwhile endeavor.*

R2.1.4:

In other places in the paper, the authors seem to have their cake and eat it too. In experiment 1, they nicely show that their algorithm can learn a 4.14:3 ratio. In fact, it could learn any ratio. So why not be clear about this in the abstract, conclusions, and throughout the paper. The clear prediction of the model is that humans can learn any rhythmic pattern equally well. Except, ... on lines 610 - 612 “However, we don’t discount the possibility of intrinsic (innate) priors which could be incorporated into the model.” This is frustrating, as the authors seem unwilling to take a theoretical stance. It seems that they don’t want to admit any limitations.

Finally, in the two paragraphs beginning on line 602, the authors clarify that it doesn't matter whether a 'bias' is 'universal' or 'learned'. If it turns out that a specific "mechanistic model" shows a universal bias, then they will just change their model to include integer ratio priors. So now I'm perplexed. Isn't it the role of a theory to make predictions about important issues like this?

I suggest one paragraph at the end that straightforwardly and clearly states the findings of this modeling work, the limitations and shortcomings, and the challenges for future research.

AR-R2.1.4: *As we stated earlier, and noting that we are not referring to innateness when referring to universality, we don't believe there is sufficient empirical evidence to warrant a firm theoretical position on what underlies the universal bias towards certain integer ratios. We think the present work contributes evidence that learning can account for this bias for existing empirical data. But given the sparsity of the empirical data and the fact that pPIPPET is not a mechanistic neural model of entrainment (see next response), we cannot discount the possibility of representational constraints stemming from the physiological level. Crucially, in future research, we propose to reconcile such constraints by extending pPIPPET (**not** changing it), by imposing constraints on the templates that can be learned and comparing the behavior of this model with the existing unconstrained version. We have made this clear in the revised manuscript (lines 603-604).*

We have renamed the final section from "Future Considerations" to "Conclusions and future considerations", and have tried to provide a clearer summary of the work (including limitations), as you proposed (lines 785-795).

R2.1.5: Suggestions for consonance with oscillatory approaches (lines 646-657) are interesting. But I don't think top-down vs bottom-up is the issue. The oscillator approach, while respecting the dynamics at the physiological level, is formulated at the behavioral level (e.g., the canonical model; Large, Almonte, & Velasco, 2010). The approach says: if you consider the dynamics of cortical oscillation and Hebbian learning at the physiological level, here are the predictions at the behavioral level. And integer ratio resonances intrinsic to coupled neural oscillation, there's really no getting around that. So, combining the Bayesian approach with the oscillator approach would be more about combining categorization and entrainment, I would think.

AR-R2.1.5: *We are pleased that you found the suggestions interesting. While the oscillator approach may be formulated at the behavioral level, we feel your description of the approach alludes to a bottom-up characterisation: "dynamics … at the physiological level" (featuring "intrinsic" integer ratio resonances) inform "predictions at the behavioral level". Of course, pPIPPET is not formulated with the same level of detail in terms of its physiological basis. We believe that the recent pre-print by Doelling et al. (2022, doi:10.1101/2022.06.18.496664) offers a useful perspective on the consequent relationship between Bayesian and oscillatory approaches – possibly, (adaptive) oscillators serve as "biologically plausible" implementations of rhythmic inferences (which, like here, can be described using Bayesian principles). Going a step further, with respect to learning, some specific (mathematical) forms of parameter optimization in hierarchical generative models have been directly related to Hebbian plasticity (Friston, 2008, PLOS Comp. Bio., p. 19; Friston, 2005, Phil. Trans. R. Soc.*

B, p. 824). We have incorporated these citations in our discussion (lines 638-650).

Characterization of the oscillation approach

R2.2.1: The revision now acknowledges neural resonance theory and its oscillation-based models. But there are some misunderstandings and mischaracterizations in the current manuscript that need to be cleared up. For example, on lines 72-74, the authors attribute the following claim: “This tendency [to ... simple integer ratios] might reflect biological constraints on rhythm processing. For example, these simple ratios offer stability for coupled neural oscillators given intrinsic mode-locking properties.”

This is almost correct. But NRT does not deal with rhythm ‘processing.’ NRT claims that the brain resonates to exogenous rhythms. The mode-locking in our theory happens at the the neural level. It does not predict a biological constraints toward perfect integer ratios at the perception or production (ie rhythm processing) level (see even Large & Kolen, 1994, Fg 3 & 6A).

Biological constraints is a (somewhat derogatory) term that has appeared in the literature, used by those who wish to incorrectly attribute limitations to NRT — such as ‘oscillator models can only predict things about isochronous stimuli’. Rather than suggest that oscillator models predict ‘biological constraints on rhythm processing’ it would be more accurate to say that ‘this tendency might reflect the physiological dynamics of oscillatory circuits.’

Additionally, (line 76) the authors claim that “Cross-frequency coupled oscillator models ... were unable to show enculturated biases for specific ratios.” But both of these papers focused on explaining integer ratio categorization, but neither attempted to explain enculturation ... and neither even included a learning rule. I would say, ‘neither offered an explanation for enculturation.’

AR-R2.2.1: *Thank you for these useful clarifications, we have followed your suggested rephrasings. We certainly did not intend our use of ‘biological constraints’ to reflect any limitations of NRT and the predictions of oscillator-based models.*

R2.2.2: Second, as mentioned above, ours is not an ‘implementation-level’ model. So (line 80) rather than say ‘enculturation effects in entrainment might be explained using such (low-level) neural mechanisms’, how about ‘.. might be explained using a model based on the dynamics of neural oscillation and Hebbian learning.’

On lines 80 - 81 "it is unclear whether these results would generalize to the cultural differences observed in iterated reproduction studies.” This conflicts with the statement on lines 654-657 “One interesting point of convergence is in the experience-dependent learning mechanisms proposed – implicit statistical learning might follow the very associative principles of Hebbian learning [68].” How about ‘it has not been shown that these results generalize ... .’

AR-R2.2.2: *We have followed both of these suggested rephrasings.*

Issues with Experiment 1:

R2.3.1: On lines 273-275, the authors state that “While the timing of stimulus events is closer to a 1:1 rhythm numerically, the stimulus events are attributed to the uneven 2:1 template as the 2:1 template is configured with a lower precision than the 1:1 template. The specificity (high precision) of the 1:1 expectations causes unevenly timed rhythms (e.g. 4:3) to be associated with the comparatively imprecise 2:1 expectations. We liken this effect to the widely observed tendency of listeners to associate uneven rhythms with a long-short pattern (i.e. 2:1).”

But aren’t the authors simply building in the behavior that they want to see, because it would not otherwise emerge from the model given the principles they have specified?

AR-R2.3.1: *As noted previously in* ***AR-R3.3.1****, we were unable to derive model parameters as in Experiments 2-3 – there is a corpus of Malian Jembe (Clayton et al. 2021, curated by Polak), but it is small in scale compared to the others used in Experiments 2-3 (from Van der Weij, 2020), which could result in underfitting. We therefore opted to manually configure models as an illustrative example. Though, as you suggest, it is possible that an alternative parameterisation would result in differing behavior, and we have explicitly considered this (with respect to* ***R3.2,*** *lines 305-310).*

R2.3.2: More importantly, in the caption to Figure 5 and in lines 300-322 the authors claim that they have ‘reproduced’ the data from Polak et al. However, if that claim is to be made, they should include the Polak data in this paper, and measure the fit quantitatively. Otherwise, they should temper the claims to something like ‘shows qualitative agreement.’

AR-R2.3.2: *We agree that ‘reproduced’ is inaccurate, and have followed this suggestion in the relevant places.*

Discussion:

R2.4.1: The authors state that “We demonstrated ... perceptual bias towards specific rhythmic patterns during iterated reproduction. This bias reflects the interval distributions of music in the training corpora, which exhibit a universal tendency for simple integer ratio rhythms alongside culture-specific biases towards rhythmic patterns deviations from simple integer ratios.”

This statement is getting closer to the type of summary that should be included. However, the statement “the interval distributions of music in the training corpora ... exhibit a universal tendency for simple integer ratio rhythms ... “ is not accurate. The interval distributions did not exhibit a universal tendency, the training corpora included only quantized (IR) ratios.

Only performed rhythms could — in principle — exhibit a universal tendency. The authors should revise this statement to reflect this.

AR-R2.4.1: *We have rephrased this, and removed the term "universal tendency". But even with quantized ratios it is important to note again that here we refer to relative distributions of simple ratios, and the training corpora featured both simple and complex ratios.*

R2.4.2: Also, the training corpora did not exhibit "culture-specific biases towards rhythmic patterns deviations from simple integer ratios." If the authors mean that the model (not the training corpora can exhibit): "culture-specific biases towards rhythmic patterns deviations from simple integer ratios," then the statement should be rewritten to say this. Also, for transparency, this paragraph should clearly state that, in fact, the model will learn whatever patterns it is trained on.

AR-R2.4.2: *We have rephrased this; and in relating these biases to the interval distributions in the training corpora, we added a reminder that the patterns learned by the model are not constrained (lines 483-487).*

R2.4.3: lines 612-614: "We have shown that present empirical results can be accounted for as a process of probabilistic learning. Presumably, musical systems serve as a proxy for priors if they incorporate innate perceptual biases and production constraints [54]." Again, I don't think this is clear. I think it means: We have shown that present empirical results can be accounted for if the musical training material consists only of integer ratio rhythms."

AR-R2.4.3: *We have clarified that the training is performed using musical corpora consisting of notated rhythms (as per* **AR-R2.1.2***).*

R2.4.4: Finally, in lines 739-742 the authors state that "Notably, the authors highlight that this model differs from oscillator-based models with limit cycle regimes, in that the striatium actively shapes SMA activity cycle-by-cycle [84, p10] – which might be better likened to a resetting drift-diffusion model [85]." First, cycle-by-cycle interaction is how coupled oscillators work, so 84 is not a well-informed counter-argument to oscillation. And [85] was published in 2017. But in 2019, Merchant (PLoS biology) showed oscillatory trajectories in medial premotor cortices (MPCs; pre-supplementary motor area [preSMA] and supplementary motor area [SMA].

AR-R2.4.4: *We have removed this comment, given our proposed implementation of pPIPPET and related discussion is not contingent on this point raised in Cannon & Patel (2021, [84]).*

Continuing problem with definition and usage of 'entrainment':

R2.5: Rather than acknowledging the physical definition of entrainment, from which the use of this term derives in this field, they have chosen to provide an empirical definition — a definition which is used in empirical work by those who are empirically investigating the phenomenon and necessarily must refrain from assuming the answer to the question they ask. As I have already commented, I don't believe this is appropriate for a theoretical paper,

in which an alternative mechanism is proposed. Or perhaps, since the model is about synchronizing a periodic process with a stimulus, it may be about the physical entrainment, in which case the authors would be claiming our understanding of the physics of entrainment to be revised.

AR-R2.5: *We think we use the term "entrainment" in the same way it is used in much of the empirical literature, and indeed we are modeling empirical results. We do not argue for a different physical definition of entrainment, and instead believe that inference processes offer a computational description of the same phenomenon -- as discussed in* ***AR2.1.5****, we explicitly state that oscillatory approaches might be wholly compatible with our model (lines 633-650). In fact, we are working on a separate manuscript demonstrating that a variant of PIPPET (compatible with pPIPPET) can be viewed mathematically as a rather specific oscillator, addressing this interesting problem, but regret that this exposition will not be available as background for the present manuscript. We have explicitly acknowledged the physical definition of entrainment and clarified our usage of the term in the Introduction (lines 14-22).*

Miscellaneous comments:

R2.6.1: In lines 24 - 37 the authors the authors state the there is a history (by the way, it is a *recent* history ... not a long tradition, perhaps this could be made clear) of simulating perception and production of rhythm as dynamically mapping between continuous auditory rhythms and discrete symbolic representations. Is this the problem the authors are trying to solve? If not, please be clear that this is a conceptual difficulty with the theory, not merely a problem with the model.

AR-R2.6.1: *Here we point out that existing probabilistic models of rhythm perception do* ***not*** *perform this dynamic mapping, as they are limited to discrete symbolic representations (lines 34-36); and that this inhibits their application to continuous auditory rhythms and real-time behavior, including enculturated biases in entrainment. We clearly state that this limitation leaves "considerable room for improvement in our computational understanding of how probabilistic temporal expectations continuously bias entrainment" (lines 36-39).*

R2.6.2: In lines 60-62 the authors state that “There was also substantial variation in the perceptual categories (rhythmic patterns) measured, which was *clearly* related to the prior music-cultural experience of participants.” This is an overstatement. This paper shows a relationship for two of the 39 participant groups. A more accurate formulation would be that they showed ‘some evidence.’

AR-R2.6.2: *This statement (in our Introduction) refers to the cited work by Jacoby et al. (2017, 2021), i.e. analysis across 39 participant groups, which we believe provides compelling evidence for culture-specific variation in more than two participant groups. Jacoby et al. (2021, pp. 12-13) draw attention to pronounced reproduction of specific (and culturally-relevant) rhythms in different groups: for a 2:2:3 rhythm, musicians in Turkey, Botswana, and Bulgaria; for a 3:3:2 rhythm, musicians from Botswana, Mali, Uruguay, Brazil*

and US/UK (jazz musicians); and for a 7:2:3 rhythm, all groups from Mali.

Reviewer #3:

R3.0: Thanks to the authors for the careful consideration of the review comments. It was an interesting read. I am overall satisfied with the changes. There were several points at which the authors considerately left the door open for future comment on my part and I do not feel so strongly about those issues as to request another round of review, or rather feel they are of interest to a rather small (but not zero!) set of readers, so I hope we'll have a chance to talk about it in the future.

AR-R3.0: *Thank you for your positive and detailed feedback in the previous round of reviews. We are delighted that you found the paper interesting, and look forward to discussing the work further. Note that page and line numbers below correspond to the version of the manuscript with tracked changes.*

R3.1: Two clarifications: AR-R3.8.18: by 'sensory updating models' I was referring simply to the class of discrete models of phase and period updating based on event timing which are swayed entirely by external events. Yes, interesting discussion, particularly regarding rhythmic re-interpretation.

AR-R3.1: *This is a helpful clarification, thank you. We completely agree with your point raised in **AR-R3.8.18**, and have explicitly noted that they resemble the case where pPIPPET is configured with metronomic expectations (lines 695-696, i.e. unlike the case where "the agent is perceptually aware of the template").*

R3.2: AR-R3.8.27 para 2: You are correct in your reading. However, I was 'looking' and comparing the _German_ and Malian group (sorry Bulgarians), perhaps biased by your later use of German folksongs to think of them as more representative of simpler meter folk. The statistical equality of Malians and Europeans at 2:1 is clearly driven by the Bulgarian respondents, while a Malian/German comparison would likely show differences at both ratios. It's a striking asymmetry present in your model between Europeans at 1:1 and 2:1, which is not seen in the (German) data. Sometimes insight can come from these details. Perhaps something worth pondering and including a sentence about if you feel you have enough information to make a meaningful comment.

AR-R3.2: *This is a good observation, thank you! Indeed, direct comparison between Malian/German and Malian/Bulgarian groups might yield different results (seemingly). We performed a very preliminary simulation with pPIPPET, qualitatively reproducing the German distribution relative to the Malian group–by doubling expectation precision (v_i) of templates in our "European" model. As you likely expected, this presents new significant differences versus the Malian model: stronger now at 1:1, $t(50)=3.22$, $p=0.002$; and now also at 2:1, $t(50)=2.46$, $p=0.016$. It would be interesting to consider if/how this relates to the difference in synchronisation consistency between the German and Bulgarian groups, as reported by*

Polak et al for an isochronous baseline (slow tempo) in Fig A1. We have added a note accordingly on how pPIPPET might account for these intra-group differences (lines 305-310).
